# Antibiotics in the Soil Environment—Degradation and Their Impact on Microbial Activity and Diversity

**DOI:** 10.3389/fmicb.2019.00338

**Published:** 2019-03-08

**Authors:** Mariusz Cycoń, Agnieszka Mrozik, Zofia Piotrowska-Seget

**Affiliations:** ^1^Department of Microbiology and Virology, School of Pharmacy with the Division of Laboratory Medicine, Medical University of Silesia, Sosnowiec, Poland; ^2^Department of Biochemistry, Faculty of Biology and Environmental Protection, University of Silesia, Katowice, Poland; ^3^Department of Microbiology, Faculty of Biology and Environmental Protection, University of Silesia, Katowice, Poland

**Keywords:** antibiotics, degradation, DT50, microbial activities, microbial community structure, antibiotic resistance genes, metagenomics, soil

## Abstract

Antibiotics play a key role in the management of infectious diseases in humans, animals, livestock, and aquacultures all over the world. The release of increasing amount of antibiotics into waters and soils creates a potential threat to all microorganisms in these environments. This review addresses issues related to the fate and degradation of antibiotics in soils and the impact of antibiotics on the structural, genetic and functional diversity of microbial communities. Due to the emergence of bacterial resistance to antibiotics, which is considered a worldwide public health problem, the abundance and diversity of antibiotic resistance genes (ARGs) in soils are also discussed. When antibiotic residues enter the soil, the main processes determining their persistence are sorption to organic particles and degradation/transformation. The wide range of DT50 values for antibiotic residues in soils shows that the processes governing persistence depend on a number of different factors, e.g., physico-chemical properties of the residue, characteristics of the soil, and climatic factors (temperature, rainfall, and humidity). The results presented in this review show that antibiotics affect soil microorganisms by changing their enzyme activity and ability to metabolize different carbon sources, as well as by altering the overall microbial biomass and the relative abundance of different groups (i.e., Gram-negative bacteria, Gram-positive bacteria, and fungi) in microbial communities. Studies using methods based on analyses of nucleic acids prove that antibiotics alter the biodiversity of microbial communities and the presence of many types of ARGs in soil are affected by agricultural and human activities. It is worth emphasizing that studies on ARGs in soil have resulted in the discovery of new genes and enzymes responsible for bacterial resistance to antibiotics. However, many ambiguous results indicate that precise estimation of the impact of antibiotics on the activity and diversity of soil microbial communities is a great challenge.

## Introduction

**Figure 1:**
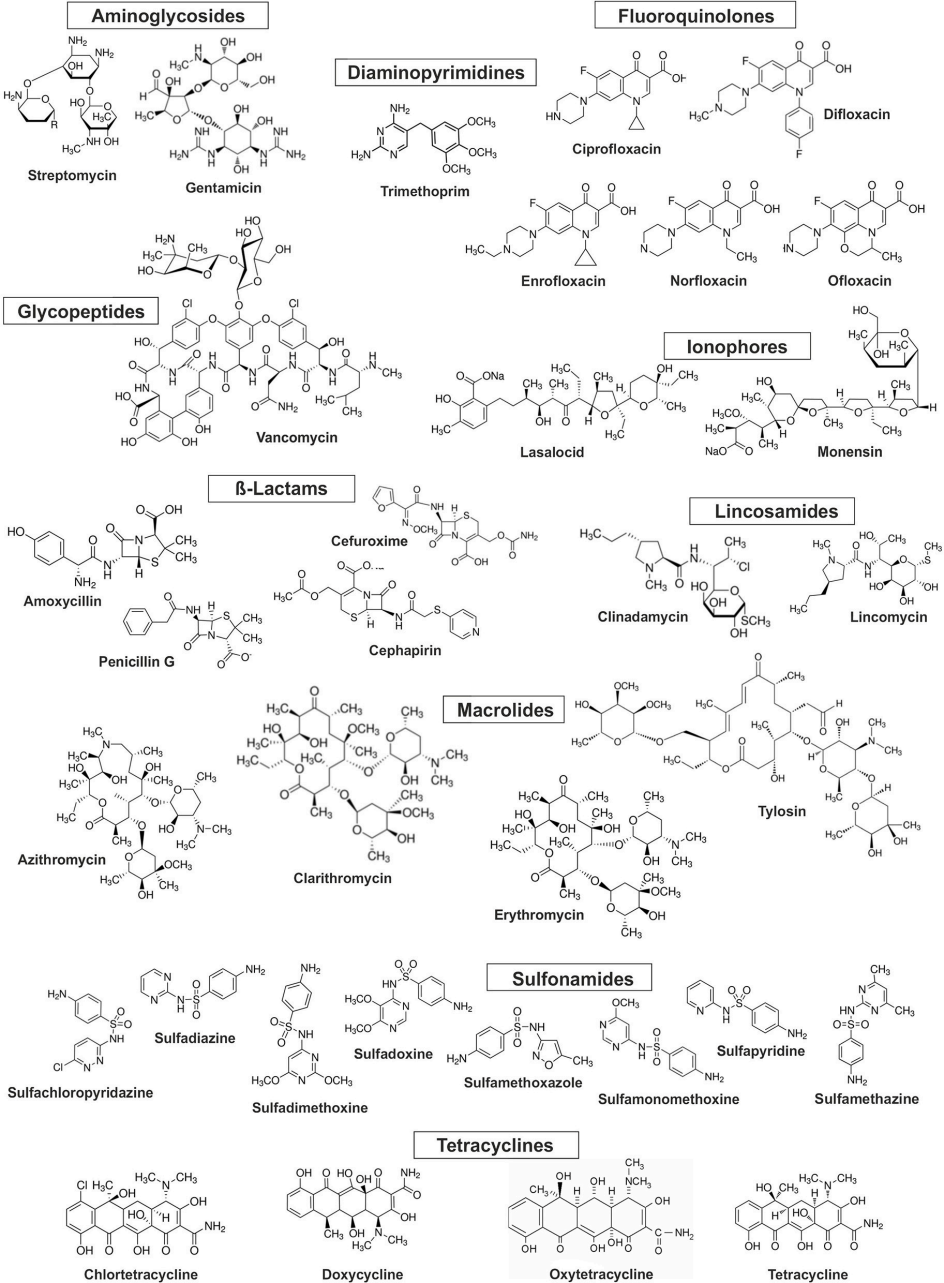
Chemical structure of the selected antibiotics.

Antibiotics are complex molecules with different functional groups in their chemical structures and are divided into several classes (Figure 1, Table 1) depending on the mechanisms of action, i.e., inhibition of cell wall synthesis, alteration of cell membranes, inhibition of protein synthesis, inhibition of nucleic acids synthesis, competitive antagonism, and antimetabolite activity (Kümmerer, 2009). Antibiotics are widely prescribed for treatment of infectious diseases in humans and animals. Moreover, they are used in livestock to increase meat production by preventing infections or outbreaks of diseases and promoting growth at a global scale. The production of antibiotics is still increasing, and the total annual usage has reached from 100,000 to 200,000 tons worldwide (Gelband et al., 2015). Between 2000 and 2015 antibiotic consumption in 76 countries around the world, expressed in defined daily doses (DDDs), increased 65% and, in 2015, reached 42 billion DDDs. Among high-income countries, the leading consumers of antibiotics in 2015 were the United States, France, and Italy. Leading consumers of antibiotics between low and middle-income countries were India, China, and Pakistan (Klein et al., 2018). It has been predicted that in 2030 global antibiotics consumption will be 200% higher than in 2015, with the greatest increase coming from low and middle-income countries. There are significant differences in trends in the antibiotic consumption in European countries. According to the Antimicrobial Consumption—Annual Epidemiological Report for 2016 published by the European Center for Disease Prevention and Control (ECDC), a statistically significant trend of increasing antibiotics usage was observed for Greece and Spain from 2012 to 2016, while over the same time period a statistically significant decreasing antibiotics usage trends were observed for Finland, Luxembourg, Norway and Sweden (ECDC, 2018). The most prescribed antibiotic classes in the US and EU are penicillins, macrolides, cephalosporins, and fluoroquinolones (CDC Centers for Disease, 2015; ECDC, 2018). More detailed information about consumption of different antibiotics in some countries of the EU and US was presented by Singer et al. (2016).

**Table 1:** Basic description and physico-chemical properties of the selected antibiotics.

**Class**	**Antibiotic**	**Chemical formula**	**Molecular weight (g/mol)**	**Water solubility (mg/L)**	**Log *K*_**OW**_**	***K*_**d**_ (L/kg)**	***K*_**OC**_ (L/kg)**
Aminoglycosides	Gentamicin	C_21_H_43_N_5_O_7_	477.6	100,000	−3.1	–	–
	Streptomycin	C_21_H_39_N_7_O_12_	581.6	12,800	−6.4	8–290	580–11,000
Diaminopyrimidines	Trimethoprim	C_14_H_18_N_4_O_3_	290.3	400	0.91	7.40	4,600
Fluoroquinolones	Ciprofloxacin	C_17_H_18_FN_3_O_3_	331.3	30,000	0.28	427–4,844	1,127–61,000
	Difloxacin	C_21_H_19_F_2_N_3_O_3_	399.4	1,330	0.89	–	–
	Enrofloxacin	C_19_H_22_FN_3_O_3_	359.4	>53.9	0.7	0.54–5,612	39–768,740
	Norfloxacin	C_16_H_18_FN_3_O_3_	319.3	177,900	−1.03	591–5,791	310
	Ofloxacin	C_18_H_20_FN_3_O_4_	361.4	10,800	0.35	1,471–4,325	44,140
Glycopeptides	Vancomycin	C_66_H_75_Cl_2_N_9_O_24_	1449.3	>1,000	−3.1	0.3–0.7	–
Ionophores	Lasalocid	C_34_H_54_O_8_	590.8	750	–	9–280	2.9–4.2
	Monensin	C_36_H_62_O_11_	670.9	0.003	5.43	0.5–65	2.1–3.8
β-Lactams	Amoxicillin	C_16_H_19_N_3_O_5_S	365.4	3,430	0.87	–	865.5
	Cephapirin	C_17_H_17_N_3_O^6^S_2_	423.5	1,030	−1.15	0.21–3.83	–
	Cefuroxime	C_16_H_16_N^4^O_8_S	424.4	145	−0.16	–	12.4–15.5
	Penicillin G	C_16_H_18_N_2_O_4_S	334.4	210	1.83	–	2.68
Lincosamides	Clindamycin	C_18_H_33_ClN_2_O_5_S	424.9	30.6	2.16	–	70
	Lincomycin	C_18_H_34_N_2_O_6_S	406.5	927	0.2	–	59
Macrolides	Azithromycin	C_38_H_72_N_2_O_12_	748.9	2.37	4.02	2.18	59,900
	Clarithromycin	C_38_H_69_NO_13_	747.9	1.7	3.16	262–400	150
	Erythromycin	C_37_H_67_NO_13_	733.9	2,000	3.06	130	10
	Tylosin	C_46_H_77_NO_17_	916.1	5,000	1.63	5.4–172,480	110–95,532
Sulfonamides	Sulfachloropyridazine	C_10_H_9_ClN_4_O_2_	284.7	8,200	0.31	0.90–3.5	41–170
	Sulfadiazine	C_10_H_10_N_4_O_2_S	250.3	77	−0.09	1.40–14	37–125
	Sulfadimethoxine	C_12_H_14_N_4_O_4_S	310.3	343	1.63	0.7–4.60	89–323
	Sulfadoxine	C_12_H_14_N_4_O_4_S	310.3	2700	0.7	0.6–4.9	1.8–31.3
	Sulfamethoxazole	C_10_H_11_N_3_O_3_S	253.3	610	0.89	0.6–4.9	1.2–94.9
	Sulfamethazine	C_12_H_14_N_4_O_2_S	278.3	1,500	0.89	0.23–206	60–208
	Sulfamonomethoxine	C_11_H_12_N_4_O_3_S	280.3	10,000	0.70	0.6–4.9	60–200
	Sulfapyridine	C_11_H_11_N_3_O_2_S	249.3	268	0.35	1.60–7.40	80–308
Tetracyclines	Chlortetracycline	C_22_H_23_ClN_2_O_8_	478.6	630	−0.62	1,280–2,386	794
	Doxycycline	C_22_H_24_N_2_O_8_	444.4	630	−0.02	–	–
	Oxytetracycline	C_22_H_24_N_2_O_9_	460.4	1,000	−0.9	417–1,026	2,872–93,317
	Tetracycline	C_22_H_24_N_2_O_8_	444.4	231	−1.19	417–1,026	400–93,320

*K_d_, distribution coefficient; K_OC_, soil organic carbon-water partitioning coefficient; K_OW_, octano-water partition coefficient*.

*Data obtained from McFarland et al. (1997); Nowara et al. (1997); Rabølle and Spliid (2000); Thiele (2000); Kümmerer (2001); Tolls (2001); Boxall et al. (2002, 2006); Hamscher et al. (2002, 2005); Thiele-Bruhn (2003); Jacobsen et al. (2004); Kay et al. (2004, 2005); Thiele-Bruhn et al. (2004); Halling-Sørensen et al. (2005); Kumar et al. (2005a, 2012); Schmitt et al. (2005); Thiele-Bruhn and Beck (2005); Sarmah et al. (2006); Martínez-Carballo et al. (2007); Sassman and Lee (2007); Stoob et al. (2007); Aust et al. (2008, 2010); Park and Choi (2008); Sukul et al. (2008); Zhang and Dong (2008); Karci and Balcioglu (2009); Kuchta et al. (2009); Li et al. (2009, 2010a,b, 2011, 2015); Muñoz et al. (2009); Conkle et al. (2010); Hu et al. (2010); Vazquez-Roig et al. (2010); Watanabe et al. (2010); Yang et al. (2010, 2016); Zhao et al. (2010); Fan et al. (2011); Lin and Gan (2011); Rosendahl et al. (2011); Zhou et al. (2011, 2013b); Leal et al. (2012); Pinna et al. (2012); Shi et al. (2012); Bak et al. (2013); Huang et al. (2013); Kang et al. (2013); Wu et al. (2013, 2014); Awad et al. (2014); Chen et al. (2014); Ho et al. (2014); Pan et al. (2014); Rutgersson et al. (2014); Van Doorslaer et al. (2014); Wegst-Uhrich et al. (2014); Gao et al. (2015); Hou et al. (2015); Liu et al. (2015); Wang and Wang (2015); DeVries and Zhang (2016); Pan and Chu (2016, 2017b); Tasho and Cho (2016); Zhang et al. (2016b, 2017a)*.

**Figure 2:**
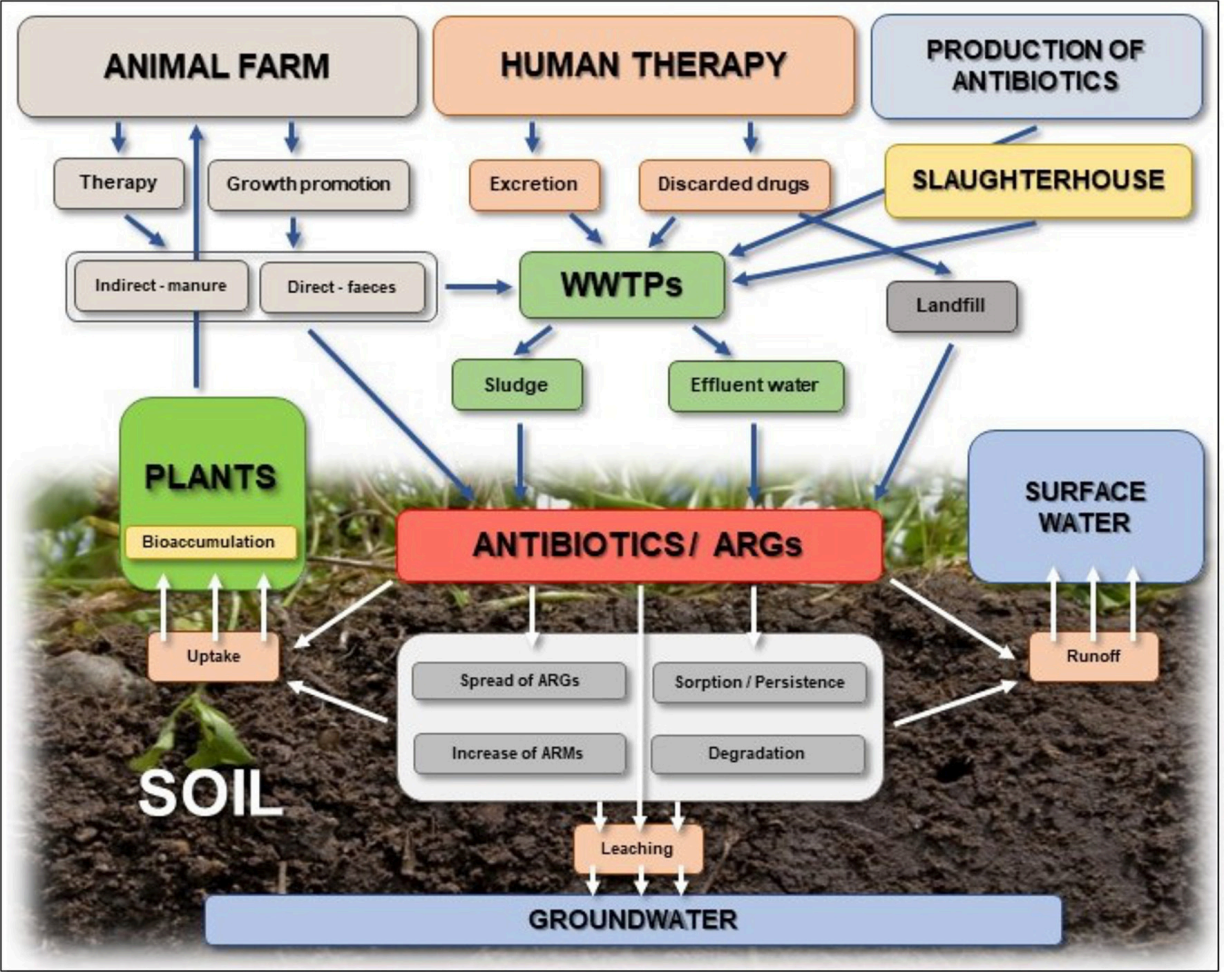
Sources and fate of antibiotics in the soil environment.

Despite their benefits, a continuous release of antibiotics into the environment and their potential adverse impact on living organisms is of great concern (Fatta-Kassinos et al., 2011; De la Torre et al., 2012; Larsson, 2014; Barra Caracciolo et al., 2015; Brandt et al., 2015). Because the majority of antibiotics are not completely metabolized in the bodies of humans and animals, a high percentage of administered drugs is discharged into water and soil through municipal wastewater, animal manure, sewage sludge, and biosolids (nutrient-rich organic materials resulting from the treatment of sewage) that are frequently used to irrigate and fertilize agricultural lands (Bouki et al., 2013; Daghrir and Drogui, 2013; Wu et al., 2014) (Figure 2). It has been reported that 75–80, 50–90, and 60% of the doses of tetracyclines, erythromycin, and lincomycin, respectively, are excreted in urine and feces (Kumar et al., 2005a; Sarmah et al., 2006). Reported antibiotic concentrations in wastewater vary significantly and range from nanograms to micrograms per mL (Gulkowska et al., 2008; Michael et al., 2013; Kulkarni et al., 2017). Though some wastewater treatment processes can degrade antibiotics, there is notable variability in antibiotic removal rates. This can be attributed to differences in treatment processes, such as nature of influent, treatment plant capacity, and the type of technology used (Forsberg et al., 2012; Wu et al., 2014).

The concentrations of antibiotic residues in manure, sewage sludge, biosolids, and soil show large variations (Table 2) and depend on the type of drug, metabolism of the drug in animals, the duration of treatment and the time of sampling relative to the treatment period. Tetracyclines have the highest concentrations and are most frequently reported antibiotic residues in manure (Pan et al., 2011; Chen et al., 2012; Massé et al., 2014). Other groups of antibiotics with considerable concentrations in manure are fluoroquinolones (Zhao et al., 2010; Van Doorslaer et al., 2014) and sulfonamides (Martínez-Carballo et al., 2007). Among the macrolide antibiotics, the highest concentration in manure was measured for tylosin (Dolliver et al., 2008). Compared to manure, biosolids contain much lower amounts of antibiotics (Jones-Lepp and Stevens, 2007) (Table 2).

**Table 2:** Maximum reported concentrations of selected antibiotics detected in manure, sewage sludge, biosolids, and soil.

**Class**	**Antibiotic**	**Concentration**	**References**
**MANURE, μg/kg**			
Fluoroquinolones	Ciprofloxacin	45,000	Zhao et al., 2010
	Enrofloxacin	1,420	
	Fleroxacin	99,000	
	Norfloxacin	225,000	
Macrolides	Tylosin	7,000–8,100	Dolliver et al., 2008; Berendsen et al., 2015
Sulfonamides	Sulfadiazine	91,000	Martínez-Carballo et al., 2007
	Sulfadimidine	20,000	
Tetracyclines	Chlortetracycline	764,000	Massé et al., 2014
	Oxytetracycline	354,000	Chen et al., 2012
	Tetracycline	98,000	Pan et al., 2011
**SEWAGE SLUDGE, μg/kg dw**			
Diaminopyrimidines	Trimethoprim	133	Göbel et al., 2005
Fluoroquinolones	Ciprofloxacin	426 (8,905)	Lillenberg et al., 2010; Li et al., 2013
Macrolides	Azithromycin	1.3–158	Göbel et al., 2005; Li et al., 2013
Sulfonamides	Sulfadimethoxine	0–20 (22.7)	Lillenberg et al., 2010; Li et al., 2013
Tetracyclines	–	8,326	Cheng et al., 2014
**BIOSOLIDS, μg/kg dw**			
Lincosamides	Lincomycin	2.6	Ding et al., 2011
Macrolides	Azithromycin	14 (6,500)	Jones-Lepp and Stevens, 2007
	Erythromycin	41 (6,500)	Kinney et al., 2006
Sulfonamides		650	US EPA, 2009
Tetracyclines	Oxytetracycline	743.6 (8,700)	US EPA, 2009; Ding et al., 2011
**SOIL, μg/kg**			
Fluoroquinolones	Ciprofloxacin	5,600	Thiele-Bruhn, 2003; Martínez-Carballo et al., 2007; Karci and Balcioglu, 2009; Hu et al., 2010; Van Doorslaer et al., 2014; Pan and Chu, 2017b
	Difloxacin	21.5	
	Enrofloxacin	1,347.6	
	Norfloxacin	2,160	
	Ofloxacin	898	
Ionophores	Monensin	0.0004	
Lincosamides	Lincomycin	0.117	
Macrolides	Enrofloxacin	22.93	Thiele-Bruhn, 2003; Leal et al., 2012; Tasho and Cho, 2016; Pan and Chu, 2017b
	Erythromycin	7.2	
	Tylosin	1,250	
Sulfonamides	Sulfachloropyridazine	52.9	Thiele-Bruhn, 2003; Dolliver et al., 2007; Karci and Balcioglu, 2009; Hu et al., 2010; Carter et al., 2014; Pan and Chu, 2017b
	Sulfadiazine	85.5	
	Sulfadimethoxine	40.4	
	Sulfadoxine	9.1	
	Sulfamethoxazole	54.5	
	Sulfamethazine	200–25,000	
	Sulfamonomethoxine	5.37	
	Sulfapyridine	5.11	
Tetracyclines	Chlortetracycline Doxycycline Oxytetracycline Tetracycline	12,900 728 50,000 2,683	Hamscher et al., 2002; Thiele-Bruhn, 2003; Karci and Balcioglu, 2009; Hu et al., 2010; Liu et al., 2016; Tasho and Cho, 2016; Pan and Chu, 2017b; Łukaszewicz et al., 2018

*dw, dry weight*.

Scientists have long been aware of potential problems from the presence of antibiotics in soil. Determined antibiotic concentrations in soil matrices have ranged from a few nanograms to milligrams per kg of soil (Table 2). The highest concentrations are usually found in areas treated with manure or used for livestock (Kay et al., 2004; Zhou et al., 2013a; Hou et al., 2015; DeVries and Zhang, 2016). The concentrations of oxytetracycline and chlortetracycline in some agricultural lands may reach extremely high levels, whereas the concentrations of ciprofloxacin, norfloxacin, and tetracycline are typically significantly lower. Accurate quantification of antibiotics and their transformation products in the soil is of the utmost importance and requires advanced analytical methods, such as high-performance liquid chromatography with tandem mass spectrometry (HPLC/MS) (Aga et al., 2016).

**Figure 3:**
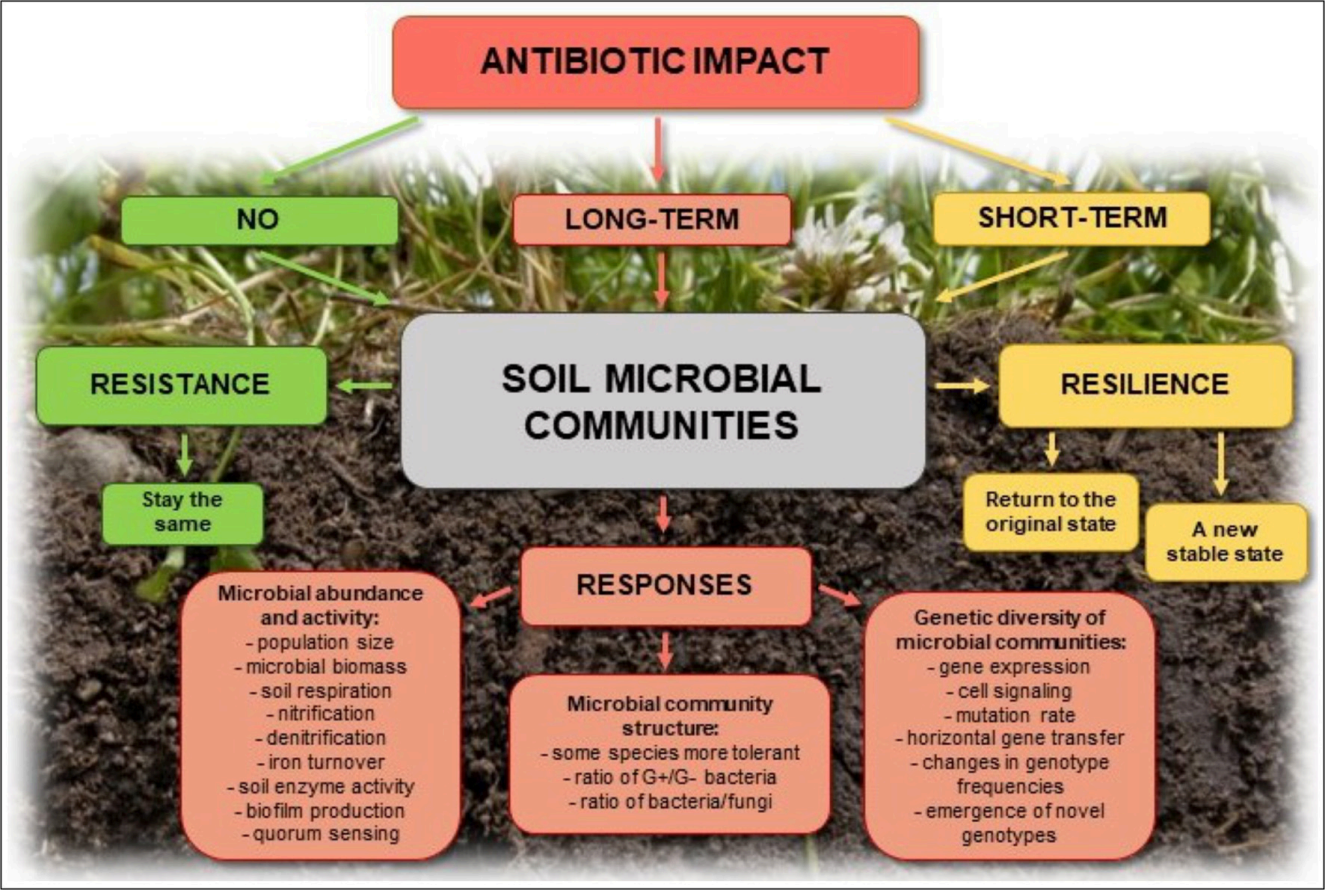
Potential effects of antibiotics on soil microbial communities and their possible responses.

Elevated concentrations of antibiotics in the soil selects for preferential outgrowth of antibiotic-resistant bacteria, which results in changes to antibiotics sensitivity of entire microbial populations (Halling-Sørensen et al., 2005; Binh et al., 2007; Ghosh and LaPara, 2007; Kyselková et al., 2013; Ma et al., 2014; Wepking et al., 2017; Atashgahi et al., 2018) (Figure 3). Even very low concentrations of antibiotics in the soil [below the minimum inhibitory concentration (MIC)], creates conditions for genetic changes in bacterial genomes and transfer of antibiotic resistance genes (ARGs) and associated mobile genetic elements (MGEs), such as plasmids, transposons, and genomic islands, between and among microbial populations (Ghosh et al., 2009; Heuer et al., 2011a,b; Du and Liu, 2012; Keen and Patrick, 2013; Tang et al., 2015; Grenni et al., 2018). In addition, co-selection and expression of resistance genes on MGEs may promote the spread of ARGs, even between distantly related bacterial species (Wellington et al., 2013). Autochthonous bacteria in soil may also represent a reservoir of resistance genes in the environment that can be transferred to the bacteria that colonize the human body (Zhang et al., 2015; Zhou et al., 2017). Such genes, e.g., tetracycline-resistance genes, were found in three different soils collected from Yunnan, Sichuan, and Tibet in China (Wang et al., 2017).

Apart from the selection of antibiotic-resistant microorganisms and the spread of resistance genes in the soil environment, antibiotics may also affect the abundance of soil microorganisms (Pinna et al., 2012; Akimenko et al., 2015; Xu et al., 2016), overall microbial activity (Schmitt et al., 2005; Brandt et al., 2009; Cui et al., 2014; Liu et al., 2015), enzyme activity (Liu et al., 2009; Wei et al., 2009; Chen et al., 2013; Ma et al., 2016), and carbon mineralization and nitrogen cycling (Thiele-Bruhn, 2005; Rosendahl et al., 2012). The impacts of antibiotics on the functional, structural and genetic diversity of soil microorganisms have also been reported (Zielezny et al., 2006; Hammesfahr et al., 2008; Unger et al., 2013; Reichel et al., 2015; Cycoń et al., 2016b; Xu et al., 2016).

The aim of this review is to evaluate recent literature on (1) the degradation of antibiotics in soil and (2) their impact on the microbial community function, (3) the structural and genetic diversity as well as (4) the abundance and diversity of ARGs in soil.

## Fate of Antibiotics in Soil

### Degradation Rate of Antibiotics in Soil

In the soil environment, antibiotics may be subject to different abiotic and/or biotic processes, including transformation/degradation (Reichel et al., 2013; Cui et al., 2014; Manzetti and Ghisi, 2014; Duan et al., 2017), sorption-desorption (Tolls, 2001; Lin and Gan, 2011; Kong et al., 2012; Leal et al., 2013; Vaz et al., 2015; Martínez-Hernández et al., 2016), uptake by plants (Kumar et al., 2005b; Dolliver et al., 2007; Grote et al., 2007; Kuchta et al., 2009; Carter et al., 2014), and runoff and transport into groundwater (Carlson and Mabury, 2006; Davis et al., 2006; Kuchta et al., 2009; Park and Huwe, 2016; Pan and Chu, 2017a) (Figure 2). Hydrolysis is generally considered one of the most important pathways for abiotic degradation of antibiotics. β-lactams are especially susceptible to hydrolytic degradation, whereas macrolides and sulfonamides are known to be less susceptible to hydrolysis (Braschi et al., 2013; Mitchell et al., 2015). Photo-degradation, which contributes to degradation of antibiotics (e.g., quinolones and tetracyclines) spread on the soil surface during application of manure and slurry to agricultural land, in another important abiotic degradation process (Thiele-Bruhn and Peters, 2007). Antibiotics may also be degraded via reductive or oxidative transformation; however, data on these processes are still scarce.

**Figure 4:**
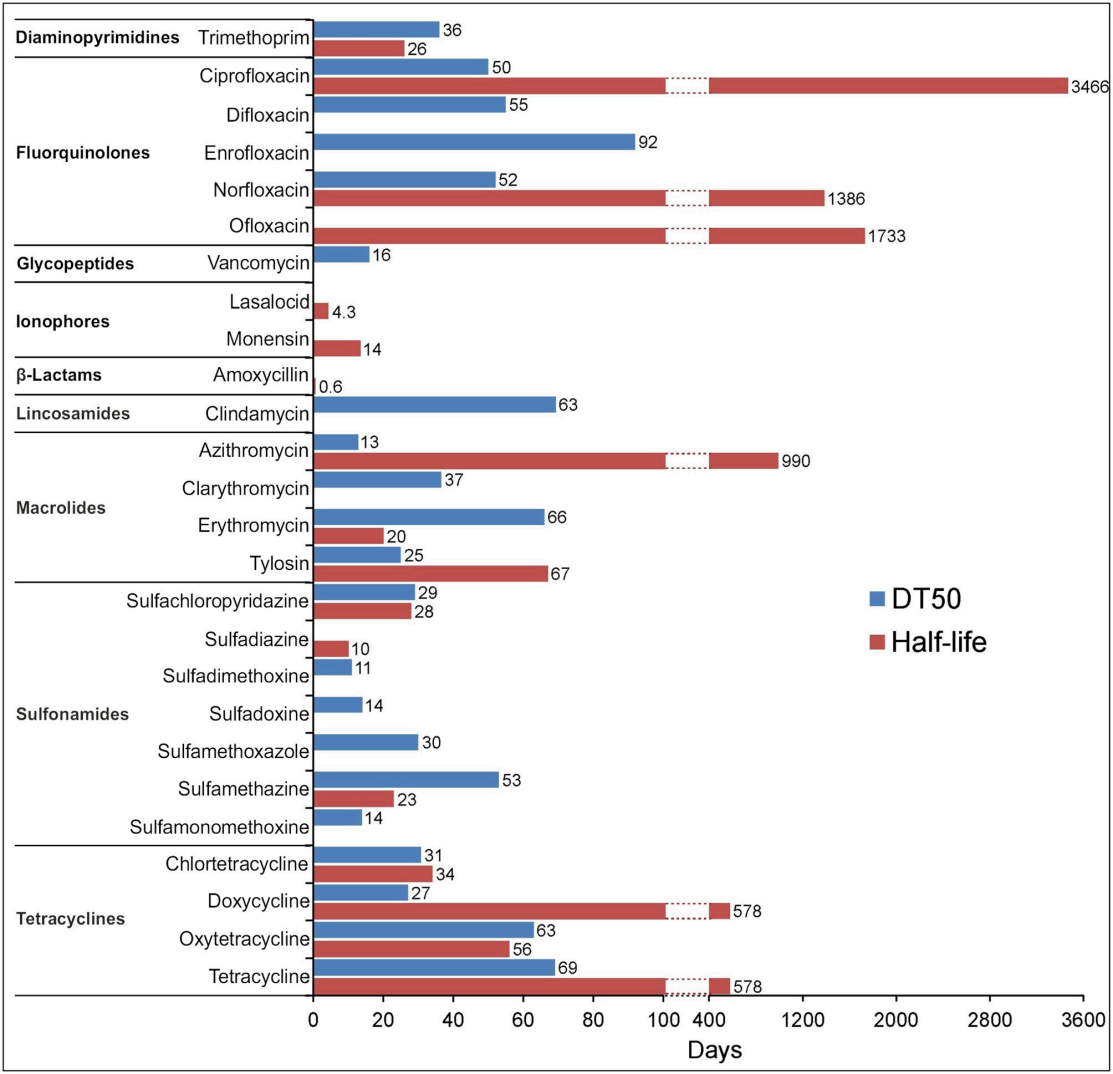
Maximum DT50 and half-life values for the selected antibiotics obtained from available degradation studies (Table 3) irrespective of the type of soil and the concentration of antibiotic used. The absence of bars in some cases means no data available.

As many authors have suggested that environmental transformation or degradation depends on the molecular structure (Figure 1) and physicochemical properties (Table 1) of antibiotics are the most important properties governing the fate of different antibiotics in soils (Thiele-Bruhn, 2003; Sassman and Lee, 2007; Crane et al., 2010; Leal et al., 2013; Awad et al., 2014; Pan and Chu, 2016; Zhang et al., 2017a). Since many abiotic and biotic factors affect the degradation of antibiotics, different groups of these pharmaceuticals differ in their rates of degradation in soils, as is evidenced by the large range of half-lives in soil, between < 1 and 3,466 days (Figure 4). For example, Braschi et al. (2013) found that amoxicillin was easily degradable, with a half-life of 0.43–0.57 days. Similarly, a study by Liu et al. (2014) revealed that chlortetracycline (10 mg/kg soil) was quickly degraded in soil with a DT50 value < 1 day. In contrast, an outdoor mesocosm study showed long-term persistence of azithromycin, ofloxacin, and tetracycline in soils, with half-lives of 408−3466, 866–1733, and 578 days, respectively (Walters et al., 2010). Detailed information about the degradation rates of antibiotics and obtained DT50 values in different soils is presented in Table 3. Notable, even for antibiotics within the same groups or, in some cases for particular antibiotics, DT50 values differ significantly. Observed differences in persistence or similar compounds are probably due to variations in soil composition, doses of antibiotics and conditions used in these studies. However, based on a review of the literature, it can be concluded that fluoroquinolones, macrolides, and tetracyclines are characterized by high DT50 or half-life values (Figure 4).

**Table 3:** Detailed data on the degradation of the selected antibiotics in soils with different characteristics.

**Antibiotic**	**Type of soil**	**Main properties of soil**	**Experimental conditions**	**Concentration (mg/kg soil)**	**Half-life/DT_**50**_ or comments**	**References**
Amoxycillin	No data	Sand 37%, silt 32%, clay 31%, pH 8.2, OC 7.7 g/kg	At field capacity conditions	0.2	Half-life 0.43 day	Braschi et al., 2013
		Sand 40%, silt 44%, clay 16%, pH 5.0, OC 21.8 g/kg			Half-life 0.57 day	
Azithromycin	Silt loam (Canada)	Sand 18%, silt 67%, clay 15%, pH 7.5, OM 3.4%, CEC 13.2 cmol+/kg	30°C, 15% moisture content, 90 days	1	DT_50_ 12.82 days (soil with a history of AZT application at 10 mg/kg soil)	Topp et al., 2016
	Sandy clay loam	Sand 53%, silt 27%, clay 20%,	Outdoor mesocosm study,	0.025	Half-life 408–990 days	Walters et al., 2010
Ciprofloxacin	(USA)	pH 5.6, OC 1.7%	average temp. 14°C, soil moisture 14.6–35.1%, 3 years, biosolids addition in a ratio 1:2	0.542	Half-life 1,153–3,466 days	
	Loam (China)	Sand 42%, silt 38%, clay 20%, pH 6.31, OM 1.29%, WHC 35%	25°C, 50% WHC, 20 days	100	100% was removed after 20 days	Ma et al., 2014
	Ustic Cambisol (China)	Sand 12%, silt 54%, clay 34%, pH 7.9, OC 36.76 g/kg, CEC 13.82 cmol+/kg	25°C, 60% WHC, 40 days	1, 5, and 50	Almost 75, 62, and 40% of antibiotic at concentrations of 1, 5, and 50 mg/kg soil, respectively, were degraded within 40 days	Cui et al., 2014
	Agricultural soil (Germany)	Sand 11%, silt 68%, clay 21%, pH 6.6, WHC 37.5%, OC 2.1%	20°C, 60% WHC, 93 days, sludge addition at 1.8 g/kg	20	0.9% of the initial concentration was mineralized	Girardi et al., 2011
Clarithromycin	Silt loam (Canada)	Sand 18%, silt 67%, clay 15%, pH 7.5, OM 3.4%, CEC 13.2 cmol+/kg	30°C, 15% moisture content, 90 days	1	DT_50_ 36.48 days (control soil—with no history of CLA application), 15.85 days (soil with a history of antibiotic application at 0.1 mg/kg soil), and 9.51 days (soil with history of antibiotic application at 10 mg/kg soil)	Topp et al., 2016
Clindamycin	Different soil types (Czech Republic)	Sand 15.85–100%, silt 0–76.74%, clay 0–14.7%, pH 5.30–8.71, OC 0.08–2.58%	20°C, 61 days	2	44–98% of the initial concentration was degraded	Koba et al., 2017
	Sandy loam (USA)	pH 6.1	30, 20, and 4°C, 30 days, manure addition	5.6	56, 12, and 0% were degraded within 30 days at 30, 20, and 4°C	Gavalchin and Katz, 1994
Chlortetracycline	Agricultural soil (China)	pH 6.92, OC 6.8 g/kg, CEC 35.2 cmol+/kg	25°C, 60% WHC, 49 days	150	DT_50_ 27.6 and 26.6–26.7 days in non-amended and manure-amended soils, respectively	Li et al., 2010a
		pH 4.55, OC 16.4 g/kg, CEC 60.0 cmol+/kg			DT_50_ 30.0 and 25.9–30.8 days in non-amended and manure-amended soils, respectively	
	Silt loam (China)	OC 18.2 g/kg, pH 5,7	25°C, 60% WHC, 22 days	1–300	DT_50_ >20 days	Liu et al., 2009
	No data (China)	Sand 42.95%, silt 43.43%, clay 13.65%, pH 7.6, OC 20.7 g/kg	25°C, 50% WHC, 45 days, DOM addition at 40 mg C/kg	10 and 100	DT_50_ < 1 and 4.7 days for 10 and 100 mg/kg, respectively	Liu et al., 2014
	Clay loam (China)	Sand 30.4%, silt 34.1%, clay 35.5%, pH 5.7, OC 18.2 g/kg, CEC 9.87 cmol+/kg	25°C, 60% WHC, 21 days	10	<30% dissipated in the first 7 days and < 55% after 21 days	Liu et al., 2012a
	Silt loam (gray brown Luvisol)	Sand 18%, silt 67%, clay 15%, pH 7.5, OM 3.4%, CEC 13.2 cmol+/kg	30°C, 15% WHC, 7 days	10	DT_50_ 3.3 days (in soil with history of exposure) and 2.8 days (in soil with no history exposure)	Topp et al., 2013
	Farm field soil (Canada)	No data	Room temp., soil moisture 20%, 47 days	1 h	Half-life 20 days in a laboratory study; half-life 21 days (24 days with manure addition) in a field study	Carlson and Mabury, 2006
	No data (China)	Sand 42.95%, silt 43.43%, clay 13.65%, pH 7.6, OC 20.7 g/kg	25°C, 50% WHC, 45 days	1, 10, and 100	DT_50_ < 1, < 1, and 5 days for 1, 10, and 100 mg/kg, respectively	Liu et al., 2015
	Inceptisol (China)	Sand 21.5%, silt 71.1%, clay 7.4%, pH 6.8, OM 3.1%, WHC 27%, CEC 10.6 cmol+/kg	25°C, 60% WHC, 35 days	1 and 100	Half-life 1.58 and 6.07 days for 10 and 100 mg/kg, respectively	Fang et al., 2016
	Loamy sand (Denmark)	Sand 75.4%, silt 10.7%, clay 11.3%, pH 6.1, OC 1.6%	Field experiment, manure addition, 155 days	0.03 and 0.05	Half-life 25 days	Halling-Sørensen et al., 2005
	Sand (Denmark)	Sand 87.6%, silt 4.8%, clay 35.2%, pH 4.3, OC 1.4%			Half-life 34 days	
Difloxacin	Luvisol (Germany)	Sand 6%, silt 78%, clay 16%, pH 6.3, OC 1.2%, CEC 11.4 cmol+/kg	21°C, 63 days, slurry addition at 40 ml/kg	0.452	Residual concentration declined to 0.258 mg/kg soil after 63 days	Reichel et al., 2013
Doxycycline	Silty clay loam (China)	Sand 9%, silt 63.7%, clay 27.3%, pH 7, OM 1%	25°C, 50–65% WHC, manure addition at 10 g/kg, 56 days	10	Almost 92% of the initial concentration was degraded during 49 days	Wang et al., 2016
	Sandy clay loam (USA)	Sand 53%, silt 27%, clay 20%, pH 5.6, OC 1.7%	Outdoor mesocosm study, average temp. 14°C, soil moisture 14.6–35.1%, 3 years, biosolids addition in a ratio 1:2	0.017	Half-life 533–578 days	Walters et al., 2010
Erythromycin	Sandy loam (Germany)	Sand 72%, silt 23%, clay 5%, pH 7.2, WHC 34.4%, OC 1.69%	20°C, water content 12%, 120 days	2	Half-life 20 days; 98% was degraded	Schlüsener et al., 2006
	Sandy loam (USA)	pH 6.1	30, 20, and 4°C, 30 days, manure addition	5.6	100, 75, and 0% were degraded within 30 days at 30, 20, and 4°C	Gavalchin and Katz, 1994
	Silt loam (Canada)	Sand 18%, silt 67%, clay 15%, pH 7.5, OM 3.4%, CEC 13.2 cmol+/kg	30°C, 15% moisture content, 90 days	1	DT_50_ 65.93 days (control soil—with no history of application), 4.36 days (soil with a history of application at 0.1 mg/kg soil), and 0.94 days (soil with a history of application at 10 mg/kg soil)	Topp et al., 2016
	Clay loam (China)	pH 6.45, OC 0.8%, WHC 50%	25°C, 70% WHC, 90 days	0.1	DT_50_ 6.4 (non-sterile soil) and 40.8 days (sterile soil) under aerobic conditions; DT_50_ 11.0 (non-sterile soil) and 57.8 days (sterile soil) under anaerobic conditions	Pan and Chu, 2016
				0.05, 0.1, and 0.2	DT_50_ 3.01–16.9 days (non-sterile soil) under aerobic conditions	
Enrofloxacin	Sandy loam (UK)	pH 5.4, OC 1.3%		10	30.3% was degraded during 56 days	Martens et al., 1996
Lasalocid	Silty clay–clay loam (Slovenia)	Sand 22.4%, Silt 49.0%, Clay 28.6%, pH 7.1, OM 4.1%, OC 2.4%	Field experiment, 21 days, manure addition at 10, 20 or 30 t/ha	3.01	Half-life 3.1 days (regardless of the treatment and soil depth)	ŽiŽek et al., 2015
	Clay loam–silty clay loam (Slovenia)	Sand 19.8%, Silt 49.6%, Clay 30.6%, pH 7.1, OM 4.1%, OC 2.4%				
	Silty clay loam (Slovenia)	Sand 15.8%, Silt 54.4%, Clay 29.8, pH 7.1, OM 4.6%, OC 2.7%				
	Sand (USA)	Clay 11%, pH 7.0, OC 0.87%, CEC 4.4 cmol+/kg	23°C, 30 days	2.1	Half-life 1.5 days	Sassman and Lee, 2007
	Clay loam (USA)	Clay 21%, pH 7.5, OC 2.91%, CEC 26.5 cmol+/kg			Half-life 3.6 days (4.3 days with manure at 20 mg/kg)	
Monensin	Farm field soil (Canada)	No data	Room temp., soil moisture 20%, 47 days	1	Half-life 13.5 days in a laboratory study; half-life 3.8 days (3.3 days with manure addition)	Carlson and Mabury, 2006
	Sand (USA)	Clay 11%, pH 7.0, OC 0.87%, CEC 4.4 cmol+/kg	23°C, 30 days	2.1	Half-life 1.3 days	Sassman and Lee, 2007
	Clay loam (USA)	Clay 21%, pH 7.5, OC 2.91%, CEC 26.5 cmol+/kg			Half-life 2 days (1.6 days with manure at 20 mg/kg)	
	Clay loam (China)	pH 6.45, OC 0.8%, WHC 50%	25°C, 70% WHC, 90 days	0.1	DT_50_ 2.91 (non-sterile soil) and 40.8 days (sterile soil) under aerobic conditions; DT_50_ 5.6 (non-sterile soil) and 53.4 days (sterile soil) under anaerobic conditions	Pan and Chu, 2016
				0.05, 0.1, and 0.2	DT_50_ 1.8–6.93 days (non-sterile soil) under aerobic conditions	
Norfloxacin	Clay loam (China)	pH 6.45, OC 0.8%, WHC 50%	25°C, 70% WHC, 90 days	0.1	DT_50_ 2.91 (non-sterile soil) and 40.8 days (sterile soil) under aerobic conditions; DT_50_ 5.6 (non-sterile soil) and 53.4 days (sterile soil) under anaerobic conditions	Pan and Chu, 2016
				0.05, 0.1, and 0.2	DT_50_ 1.8–6.93 days (non-sterile soil) under aerobic conditions	
	Silty clay loam (China)	Sand 9%, silt 63.7%, clay 27.3%, pH 7, OM 1%	25°C, 50–65% WHC, manure addition at 10 g/kg, 56 days	10	Almost 47% of initial concentration was degraded within 49 days	Wang et al., 2016
	Acidic Soil (China)	Sand 29%, silt 39%, clay 32%, pH 4.3, OM 2.4%, CEC 9.5 cmol+/kg	25°C, 50% WHC, 42 days	5, 10, and 30	Half-life 31, 48, and 62 days (without manure) for 5, 10, and 30 mg/kg, respectively; half-life 24–39 days (with manure 3−9%) for 10 mg/kg	Yang et al., 2012
	Sandy clay loam (USA)	Sand 53%, silt 27%, clay 20%, pH 5.6, OC 1.7%	Outdoor mesocosm study, average temp. 14°C, soil moisture 14.6–35.1%, 3 years biosolids addition in a ratio 1:2	0.045	Half-life 990–1,386 days	Walters et al., 2010
Ofloxacin				0.470	Half-life 866–1,733 days	
Oxytetracycline	Soil from a wheat field (China)	pH 8.45, OM 19.13 g/kg, CEC 14.84 cmol+/kg, OTC 1.65 mg/kg	20–25°C day/15°C night, 70% WHC, 60 days	300	85.6 and 87.3% were degraded in soil with compost (10%) and compost (10%) + biochar (2%), respectively	Duan et al., 2017
	Agricultural soil (China)	pH 6.92, OC 6.8 g/kg, CEC 35.2 cmol+/kg	25°C, 60% WHC, 49 days	150	DT_50_ 30.2 and 38.2–39.7 days in non-amended and manure-amended soils, respectively	Li et al., 2010a
		pH 4.55, OC 16.4 g/kg, CEC 60.0 cmol+/kg			DT_50_ 39.4 and 35.9–41.3 days in non-amended and manure-amended soils, respectively	
	Sandy loam (UK)	Sand 69–80%, silt 6–21%, clay 4–10%, pH 6.2–6.6, OC 1.3%	Field experiment, 127 days	0.3	DT_50_ 21.7 days	Blackwell et al., 2007
	Sandy loam (USA)	OM 0.92%, pH 7.2	25°C, moisture 20%, 62 days	50	Half-life 33 and 56 days in manure-amended and non-amended soils, respectively	Wang and Yates, 2008
	Alfisol (China)	Sand 7.7%, silt 77.5%, clay 14.8%, pH 6.24, OM 2.4%, CEC 12.3 cmol+/kg, OTC 37.3 μg/kg	25°C, 60% WHC, 120 days	1–30	86.6, 89.6, 93.7, and 95.4% of antibiotic at concentrations of 1, 3.6, 10, and 30 mg/kg soil, respectively, were degraded within 120 days	Ma et al., 2016
Penicillin G	Sandy loam (USA)	pH 6.1	manure addition	5.6	No degradation within 30 days	Gavalchin and Katz, 1994
Sulfachloropyridazine	Silt Loam (USA)	Sand 19.9%, silt 56.6%, clay 23.6%, pH 7.5, OC 1.8	25°C, 40 days	1, 10, and 100	Half-life 20, 20, and 22 days (non-sterile soil) for 1, 10, and 100 mg/kg, respectively; half-life 68 days (sterile soil) for 10 mg/kg	Accinelli et al., 2007
	Sand (USA)	Sand 93.5%, silt 2.7%, clay 3.8%, pH 7.2, OC 0.94%			Half-life 27, 26, and 28 days (non-sterile soil) for 1, 10, and 100 mg/kg, respectively; half-life 71 days (sterile soil) for 10 mg/kg	
	Sandy loam (UK)	Sand 69–80%, silt 6–21%, clay 4–10%, pH 6.2–6.6, OC 1.3%	Field experiment, 127 days	0.65	DT_50_ 3.5 days	Blackwell et al., 2007
	Clay loam (UK)	Sand 42.63%, silt 32.26%, clay 25.11%, pH 6.8, OC 2.2%,	Field experiment	0.2	DT_50_ 29 days	Kay et al., 2004
Sulfadimethoxine	Silt loam (USA)	Sand 8.0%, silt 65.1%, clay 26.9%, pH 5.54, OC 1.44%, moisture 1.8%	25°C, 70 days, manure addition at 5%	1, 25, 50, and 100	DT_50_ 3, 5.8, 6.8, and 11 days for 1, 25, 50, and 100 mg/kg, respectively	Wang et al., 2006
Sulfadiazine	Luvisol (Germany)	Sand 60 g/kg, silt 220 g/kg, clay 30 g/kg, pH 6.3, OC 12.2 g/kg,	10°C, 30% WHC, 218 days	2.2	DT_50_ 19 days (CaCl_2_ fraction), 24 days (MeOH fraction), and 290 days (residual fraction)	Förster et al., 2009
	Cambisol (Germany)	Sand 750 g/kg, silt 780 g/kg, clay 160 g/kg, pH 6.0, OC 9.9 g/kg		2.7	DT_50_ 15 days (CaCl_2_ fraction), 13 days (MeOH fraction), and 490 days (residual fraction)	
	Agricultural soil (China)	Sand 36.96%, silt 58.76%, clay 4.28%, pH 7.6, OC 4.71 g/kg, CEC 7.0 cmol+/kg	21°C, 49 days, WHC 60%, manure addition at 4%	4, 10, and 20	DT_50_ 8.48, 8.97, and 10.22 days (non-sterile soil), and 30.09, 26.55, and 21.21 days (sterile soil) for 4, 10, and 20 mg antibiotic/kg, respectively	Zhang et al., 2017a
	Loamy sand (Germany)	Sand 73.3%, silt 23.1%, clay 3.6%, pH 5.5, OC 1%	10°C, 50% WHC, manure addition at 40 mg/g, 61 days	10 and 100	DT_50_ < 1 and 8.5 days for 10 and 100 mg/kg, respectively	Hammesfahr et al., 2008
	Silt loam (Germany)	Sand 6.4%, silt 78.2%, clay 15.4%, pH 7.2, OC 2.1%			DT_50_ < 1 and 5.6 days for 10 and 100 mg/kg, respectively	
	Endogleyic Cambisol (Germany)	Sand 73.3%, silt 23.1%, clay 3.6%, pH 4.8, OC 1%	10°C, 50% WHC, manure addition at 20, 40, and 80 g/kg, 32 days	10 and 100	At day 1, the recovery rate ranged between 27 and 45%; on day 32 15−18% were extracted in higher treatment and 7−10% in the lower treatment	Hammesfahr et al., 2011
	Luvisol (China)	Sand 58.4%, silt 21.7%, clay 19.9%, pH 6.24, OM 3.56%, CEC 5.38 cmol+/kg	25°C, 25% WHC, manure addition at 40 mg/kg, Cu addition at 0, 20, and 200 mg/kg, 28 days	10 and 100	Degradation rate constant *k* (day) 0.115, 0.108, 0.087, and 0.041, 0.035, 0.028 at 10 mg/kg + 0, 20, and 200 mg Cu/kg, and antibiotic at 100 mg/kg + 0, 20, and 200 mg Cu/kg, respectively	Xu et al., 2016
	Luvisol (Germany)	Sand 6%, silt 78%, clay 16%, pH 6.3, OC 1.2%, CEC 11.4 cmol+/kg	21°C, 63 days, slurry addition at 40 ml/kg	0.256	< 0.002 mg antibiotic/kg soil was detected within 63 days	Reichel et al., 2013
	Silty loam (Germany)	Sand 4.3%, silt 82.9%, clay 12.8%, pH 6.7, OC 1%	60 days	0.71	DT_50_ 4.8 days	Sittig et al., 2014
	Loamy sand (Germany)	Sand 69.7%, silt 26.3%, clay 4%, pH 5.7, OC 0.9%		0.68	DT_50_ 8.6 days	
Sulfamonomethoxine	Loam (China)	Sand 42%, silt 38%, clay 20%, pH 6.31, OM 1.29%, WHC 35%	25°C, 50% WHC, 20 days	100	71.8% was removed within 20 days	Ma et al., 2014
Sulfamethoxazole	Sand (USA)	Sand 91%, silt 5%, clay 4%, pH 9.23, OC 0.16%, CEC 8.2 cmol+/kg	21°C, 75% WHC, 84 days	0.04	Half-life 11.4 (non-sterile soil) and 58.7 days (sterile soil) under aerobic conditions; half-life 18.3 (non-sterile soil) under anaerobic conditions	Lin and Gan, 2011
	Medium loam (USA	Sand 91%, silt 5%, clay 4%, pH 9.23, OC 0.16%, CEC 8.2 cmol+/kg			Half-life 9.0 and 15.3 days (non-sterile soil) under aerobic and anaerobic conditions, respectively	
	Silt loam (China)	OC 18.2 g/kg, pH 5,7	25°C, 60% WHC, 22 days	1–300	DT_50_ 2–5 days	Liu et al., 2009
	Clay loam (China)	Sand 30.4%, silt 34.1%, clay 35.5%, pH 5.7, OC 18.2 g/kg, CEC 9.87 cmol+/kg	25°C, 60% WHC, 21 days	10	More than 90% dissipated in the first 7 days	Liu et al., 2012a
	Silty clay loam (China)	Sand 9%, silt 63.7%, clay 27.3%, pH 7, OM 1%	25°C, 50–65% WHC, manure addition at 10 g/kg, 56 days	10	Almost 80% of initial concentration was degraded during 49 days	Wang et al., 2016
	Silt loam topsoil (TS)/subsoil (SS) (New Zealand)	Sand 9%/12.3%, silt 54%/62.8%, clay 37%/24.9%, pH 6.7/5.7, OC 5%/0.	25°C, 60% WHC, 36 days	0.5	DT_50_ 11 (TS) and 34.7 (SS) days (sterile soil); DT_50_ 9.2 (TS) and 11.8 (SS) days (non-sterile soil)	Srinivasan and Sarmah, 2014
	Clay loam topsoil (TS) and subsoil (SS) (New Zeland)	Sand 13.7%/13.4%, silt 51%/40.3%, clay 30.4%/46.2%, pH 5.8/5.1, OC 4%/0.8%			DT_50_ 13 (TS) and 22.4 (SS) days (sterile soil); DT_50_ 4.3 (TS) and 4.2 (SS) days (non-sterile soil)	
	Silt loam topsoil (TS) and subsoil (SS) (New Zeland)	Sand 34%, silt 48%, clay 17%, pH 5.7/6.6, OC 8.2%/1.7%			DT_50_ 18.1 (TS) and 22.7 (SS) days (sterile soil); DT_50_ 13.3 (TS) and 12.4 (SS) days (non-sterile soil)	
	Loamy sand (Netherlands)	Sand 78.9%, silt 10.4%, clay 7%, pH 4.9, OC 3.7%	25°C, 35% WHC, 5 weeks	1–500	Initial concentrations were reduced to 153, 1.5, and 0.04 mg/kg soil at 500, 20, and 1 mg/kg soil within 5 weeks	Demoling et al., 2009
	Different soil types (Czech Republic)	Sand 15.85–100%, silt 0–76.74%, clay 0–14.7%, pH 5.30–8.71, OC 0.08–2.58%	20°C, 61 days	2	25–99% of initial concentration was degraded	Koba et al., 2017
	Agricultural soil (China)	Sand 36.96%, silt 58.76%, clay 4.28%, pH 7.6, OC 4.71 g/kg, CEC 7.0 cmol+/kg	21°C, 49 days, WHC 60%, manure addition at 4%	4, 10, and 20	DT_50_ 13.68, 10.28, and 10.81 days (non-sterile soil), and 22.99, 33.24, and 22.79 days (sterile soil) for 4, 10, and 20 mg/kg, respectively	Zhang et al., 2017a
Sulfamethazine	Silt Loam (USA)	Sand 19.9%, silt 56.6%, clay 23.6%, pH 7.5, OC 1.8	25°C, 40 days	1, 10, and 100	Half-life 17, 18, and 16 days (non-sterile soil) for 1, 10, and 100 mg/kg, respectively; half-life 78 days (sterile soil) for 10 mg/kg	Accinelli et al., 2007
	Sand (USA)	Sand 93.5%, silt 2.7%, clay 3.8%, pH 7.2, OC 0.94%			Half-life 22, 23, and 23 days (non-sterile soil) for 1, 10, and 100 mg/kg, respectively; half-life 77 days (sterile soil) for 10 mg/kg	
	Silt loam (China)	OC 18.2 g/kg, pH 5,7	25°C, 60% WHC, 22 days	1–300	DT_50_ 2–5 days	Liu et al., 2009
	Silt loam (Canada)	Sand 18%, silt 67%, clay 15%, pH 7.5, OM 3.4%, CEC 13.2 cmol+/kg	30°C, 15% WHC, 7 days	100 ng/g SMZ + 10,000 dpm/g [U-phenyl-14C]-SMZ	DT_50_ 1.3 days (in soil with history of exposure) and 5.3 days (in soil with no history of exposure)	Topp et al., 2013
	Silt loam (Korea)	pH 6.0, OM 2.36%	25°C, 70% WHC, 56 days, poultry manure addition (1%)	20 and 100	The concentration of antibiotic at 100 mg/kg was 255.5 and 129.8 μg/kg while at 20 mg/kg was 62.1 and 31.5 μg/kg at the beginning and on day 56, respectively.	Awad et al., 2016
	Clay loam (China)	pH 6.45, OC 0.8%, WHC 50%	25°C, 70% WHC, 90 days	0.1	DT_50_ 24.8 (non-sterile soil) and 49.5 days (sterile soil) under aerobic conditions; DT_50_ 34.7 (non-sterile soil) and 57.8 days (sterile soil) under anaerobic conditions	Pan and Chu, 2016
				0.05, 0.1, and 0.2	DT_50_ 16.90–53.31 days (non-sterile soil) under aerobic conditions	
	Sand (Australia)	Sand 89%, silt 3%, clay 8%, pH 6.25, moisture 0.6%, OC 1%, CEC 5.2 cmol+/kg	23°C (day) and 15°C (night), 60% WHC, 40 days		DT_50_ 0.99 days	Carter et al., 2014
Streptomycin	Sandy loam (USA)	pH 6.1	Manure addition	5.6	No degradation within 30 days	Gavalchin and Katz, 1994
Tetracycline	Agricultural soil (China)	pH 6.92, OC 6.8 g/kg, CEC 35.2 cmol+/kg	25°C, 60% WHC, 49 days	150	DT_50_ 20.9 and 26.7–29.1 days in non-amended and manure-amended soils, respectively	Li et al., 2010a
		pH 4.55, OC 16.4 g/kg, CEC 60.0 cmol+/kg			DT_50_ 21.7 and 20.6–26.2 days in non-amended and manure-amended soils, respectively	
	Silt loam (China)	OC 18.2 g/kg, pH 5,7	25°C, 60% WHC, 22 days	1–300	DT_50_ > 20 days	Liu et al., 2009
	Sandy clay loam (USA)	Sand 53%, silt 27%, clay 20%, pH 5.6, OC 1.7%	Outdoor mesocosm study, average temp. 14°C, soil moisture 14.6–35.1%, 3 years, biosolids addition in a ratio 1:2	0.021	Half-life 578 days	Walters et al., 2010
	Loam (China)	Sand 42%, silt 38%, clay 20%, pH 6.31, OM 1.29%, WHC 35%	25°C, 50% WHC, 20 days	100	100% was removed within 20 days	Ma et al., 2014
	Clay loam (China)	pH 6.45, OC 0.8%, WHC 50%	25°C, 70% WHC, 90 days	0.1	DT_50_ 31.5 (non-sterile soil) and 57.8 days (sterile soil) under aerobic conditions; DT_50_ 43.3 (non-sterile soil) and 86.6 days (sterile soil) under anaerobic conditions	Pan and Chu, 2016
				0.05, 0.1, and 0.2	DT_50_ 14.1–69.3 days (non-sterile soil) under aerobic conditions	
Trimethoprim	Sand (USA)	Sand 91%, silt 5%, clay 4%, pH 9.23, OC 0.16%, CEC 8.2 cmol+/kg	21°C, 75% WHC, 84 days	0.04	Half-life 26.0 and 26.1 days (non-sterile soil) under aerobic and anaerobic conditions, respectively	Lin and Gan, 2011
	Silt loam (China)	OC 18.2 g/kg, pH 5,7	25°C, 60% WHC, 22 days	1–300	DT_50_ 2–5 days	Liu et al., 2009
	Different soil types (Czech Republic)	Sand 15.85–100%, silt 0–76.74%, clay 0–14.7%, pH 5.30–8.71, OC 0.08–2.58%	20°C, 61 days	2	13–84% of initial concentration was degraded	Koba et al., 2017
	Sandy loam (USA)	Sand 60%, silt 22%, clay 18%, pH 7, WHC 15%, OM 1.6%	20°C, 30 days	50	Half-life 7–8 days	Hu and Coats, 2007
	Sandy loam (Germany)	Sand 72%, silt 23%, clay 5%, pH 7.2, WHC 34.4%, OC 1.69%	20°C, water content 12%, 120 days	2	Half-life 8 days; 100% was degraded	Schlüsener et al., 2006
	Sandy loam (USA)	pH 6.1	30, 20, and 4°C, 30 days, manure addition	5.6	100, 100, and 60% were degraded during 30 days at 30, 20, and 4°C	Gavalchin and Katz, 1994
	Sand (Denmark)	Sand 90.7%, silt 2.8%, clay 4.1%, pH 6.8, WHC 15%, OC 1.2%	25°C, 15% WHC, 33 days	2,000	100% of TYL was degraded within13 days	Westergaard et al., 2001
Tylosin	Silt loam (Canada)	Sand 18%, silt 67%, clay 15%, pH 7.5, OM 3.4%, CEC 13.2 cmol+/kg	30°C, 15% WHC, 7 days	10	DT_50_ 2 days (in soil with a history of exposure) and 10.2 days (in soil with no history exposure)	Topp et al., 2013
	Farm field soil (Canada)	No data	Room temp., soil moisture 20%, 47 days	1	Half-life 4.4 days in a laboratory study; half-life 6.1 days (4.5 days with manure addition) in a field study	Carlson and Mabury, 2006
	Loamy sand (Denmark)	Sand 75.4%, silt 10.7%, clay 11.3%, pH 6.1, OC 1.6%	Field experiment, manure addition, 155 days	0.03 and 0.05	Half-life 67 days	Halling-Sørensen et al., 2005
	Sand (Denmark)	Sand 87.6%, silt 4.8%, clay 35.2%, pH 4.3, OC 1.4%			Half-life 49 days	
	Silt loam (China)	OC 18.2 g/kg, pH 5.7	25°C, 60% WHC, 22 days	1–300	DT_50_ 8 days	Liu et al., 2009
	Sand (Denmark)	Sand 90.7%, silt 2.8%, clay 4.1%, pH 6.8, WHC 15%, OC 1.2%	25°C, 60 days	2,000	Completely dissipated within 13 days; all degradation products disappeared after 17 days	Müller et al., 2002
	Sand (Denmark)	Sand 87.6%, silt 4.8%, clay 5.2%, pH 6.3, OC 1.4%	Manure addition	100	50% was degraded within 4.2 days	Ingerslev and Halling-Sørensen, 2001
	Sandy loam (Denmark)	Sand 75.4%, silt 10.8%, clay 11.3%, pH 6.8, OC 1.6%			50% was degraded within 5.7 days	Ingerslev and Halling-Sørensen, 2001
Vancomycin	Sandy loam (Poland)	Sand 67%, silt 24%, clay 9%, pH 6.9, WHC 43%, OC 1.6%, CEC 10 cmol/kg	22°C, 50% WHC, 90 days	1 and 10	DT_50_ 16 days; dissipation was independent of the concentration used.	Cycoń et al., 2018

*CEC, cation exchange capacity; DOM, dissolved organic matter; OC, organic carbon; OM, organic matter; URE, urease; WHC, water holding capacity*.

The sorption coefficient (K_oc_) is a very important parameter for estimating environmental distribution and environmental exposure level of antibiotics. As was proposed by Crane et al. (2010), antibiotics with values of K_oc_ > 4,000 l/kg are non-mobile and degradable to a very low degree in soils (very persistent) and the length of time needed for the degradation of 50% of an initial dose is >60 days. In contrast, antibiotics characterized by values of K_oc_ < 15 l/kg are highly mobile and are more easily degraded; these can be classified as compounds with low persistence in soils (DT50 < 5 days). The relatively low persistence of some antibiotics in soils may be related to their low affinity to various organic and non-organic soil components. In contrast, due to their properties tetracyclines, fluoroquinolones, macrolides, and sulfonamides, bind strongly to soil components and form stable residues. For example, Hammesfahr et al. (2008) showed that the recovery rate of sulfadiazine on day 1 of an experiment was 27 and 45% of the initial antibiotic dosages of 10 and 100 mg/kg soil, respectively. Similarly, sulfamethazine applied at concentrations of 20 and 100 mg/kg soil strongly absorbed to soil components and measured concentrations of antibiotic were 62.1 and 31.5 μg/kg soil at the beginning of the experiment and, 255.5 and 129.8 μg/kg soil on day 56, respectively (Awad et al., 2016). Due to the binding of antibiotics to soil particles or the occurrence in a form of complexes, sorbed antibiotics often cannot be detected and may lose their antibacterial activity (Kümmerer, 2009). The adsorption and desorption of antibiotics are also associated with other soil parameters, such as pH and water content. For example, sulfonamides show a decrease in sorption with an increase of soil pH, whereas the binding of macrolides by soil components increases with a decrease of pH. In the first case, the sorption behavior is consistent with changes in the fraction of ionization of the sulfonamide as it converts from its cationic form to the neutral and anionic forms. The behavior of sulfonamides contrasts with tetracyclines and fluoroquinolones, which interact with soil primarily through cation exchange, surface complexation and cation bridging sorption mechanisms. In general, decreases in pH resulted in increased sorption of the cationic forms of antibiotics, suggesting that electrostatic interactions are the favored sorption mechanism for sulfonamides and macrolides (Schauss et al., 2009; Hammesfahr et al., 2011; Kong et al., 2012; Teixidó et al., 2012; Sittig et al., 2014; Wegst-Uhrich et al., 2014; Fernández-Calviño et al., 2015; Wang and Wang, 2015;Liu et al., 2017).

In addition to abiotic processes, microbial degradation may contribute to disappearance of antibiotics in soil. Some bacteria that degrade antibiotics have been isolated from antibiotics-contaminated soils. For example, strains belonging to the genera *Microbacterium* (Topp et al., 2013), *Burkholderia* (Zhang and Dick, 2014), *Stenotrophomonas* (Leng et al., 2016), *Labrys* (Mulla et al., 2018), *Ochrobactrum* (Zhang et al., 2017b; Mulla et al., 2018), and *Escherichia* (Mulla et al., 2018; Wen et al., 2018) were capable of degrading sulfamethazine, penicillin G, tetracycline, erythromycin and doxycycline in liquid cultures, respectively. Other bacteria belonging to the genera *Klebsiella* (Xin et al., 2012), *Acinetobacter, Escherichia* (Zhang et al., 2012), *Microbacterium* (Kim et al., 2011), *Labrys* (Amorim et al., 2014), and *Bacillus* (Rafii et al., 2009; Erickson et al., 2014) that were capable of degrading chloramphenicol, sulfapyridine, sulfamethazine, ciprofloxacin, norfloxacin, and ceftiofur have been isolated from patients, sediments, sludge, animal feces, and seawater. In particular, a *Microbacterium* sp. exhibited degradation of sulfamethazine in soil and, when introduced into agricultural soil, increased the mineralization of that antibiotic by 44–57% (Hirth et al., 2016). The central role of microorganisms in antibiotic degradation or transformation in soil has been confirmed by results of many studies carried out in sterile and non-sterile soils. As depicted in Table 3, the half-life or DT50 values of antibiotics were much lower in soils with autochthonous microorganisms compared to those obtained from sterilized soils. For example, Pan and Chu (2016) showed that when applied to soil at a dosage of 0.1 mg/kg soil, erythromycin disappeared faster in non-sterile soil compared to sterile soil, with DT50 of 6.4 and 40.8 days, respectively. Sulfachloropyridazine applied at 10 mg/kg soil was degraded almost three times faster in soils with autochthonous microorganisms (half-life 20–26 days) compared to sterile soils (half-life 68–71 days) (Accinelli et al., 2007). Zhang et al. (2017a) also showed that microbial activity plays a major role in the biotransformation of sulfadiazine in soil s, with a DT50 of 8.48, 8.97, and 10.22 days (non-sterile soil) and 30.09, 26.55, and 21.21 days (sterile soil) for concentrations of 4, 10, and 20 mg/kg soil, respectively. A similar phenomenon has also been observed for other antibiotics, such as norfloxacin (Pan and Chu, 2016), sulfamethoxazole (Lin and Gan, 2011; Srinivasan and Sarmah, 2014; Zhang et al., 2017a), sulfamethazine (Accinelli et al., 2007; Pan and Chu, 2016), and tetracycline (Lin and Gan, 2011; Pan and Chu, 2016).

Special attention should be paid to the analytical methods for extraction and determination of antibiotics residue when evaluating and comparing degradation experiments. Some techniques may not always be capable of differentiating between degradation and sorption, and insufficient or improper extraction procedures may result in incorrect interpretation of the fate of antibiotics in the soil as antibiotics that are tightly bound to soil particles could be erroneously considered to have been transformed or degraded.

### Factors Affecting the Degradation of Antibiotics in Soil

Degradation of antibiotics depends not only on the catabolic activity of soil microorganisms, but also, to a large extent, on the properties of soil, i.e., organic matter content, pH, moisture, temperature, oxygen status, and soil texture (Table 3). For example, Li et al. (2010a) observed differences in the degradation rate of oxytetracycline in two agricultural soils with different characteristics. The calculated DT50 for this antibiotic reached values of 30.2 and 39.4 days for soils with a low or high organic carbon content, respectively. Soil type was also found to significantly affect antibiotics degradation, as demonstrated by Koba et al. (2017) who studied the degradation rate of clindamycin, sulfamethoxazole, and trimethoprim in 12 different soils. The authors characteristics, 44–98, 25–99, and 13–84% of clindamycin, sulfamethoxazole and trimethoprim (2 mg/kg soil), respectively, were degraded within 61 days during the experiment. A similar dependence of the rate of antibiotics degradation on soil type of has also been found for chlortetracycline (Halling-Sørensen et al., 2005; Li et al., 2010a), sulfachloropyridazine (Accinelli et al., 2007), sulfadiazine (Förster et al., 2009; Sittig et al., 2014), sulfamethoxazole (Accinelli et al., 2007; Lin and Gan, 2011; Srinivasan and Sarmah, 2014), and tetracycline (Li et al., 2010a). Another study revealed a significant decrease in the rate of chlortetracycline, erythromycin, and tylosin degradation introduced at a dosage of 5.6 mg/kg soil into a sandy loam soil. These degradation experiments showed that 56, 12, and 0%, 100, 75, and 0%, or 100, 100, and 60% of the initial concentration of chlortetracycline, erythromycin, and tylosin were degraded at temperatures of 30, 20, and 4°C, respectively, within 30 days (Gavalchin and Katz, 1994). In addition, Srinivasan and Sarmah (2014) showed that a lower temperature resulted in a reduced decomposition rate of sulfamethoxazole, irrespective of the soil depth. Some published data have also shown the influence of oxygen in soils on the degradation of antibiotics. For example, Pan and Chu (2016) showed that the half-lives for erythromycin, norfloxacin, sulfamethazine, and tetracycline, all of which had been applied at 0.1 mg/kg soil, increased from 6.4, 2.9, 24.8, and 31.5 days under aerobic conditions to 11.0, 5.6, 34.7, and 43.3 days under anaerobic conditions, respectively. Anaerobic conditions caused an increase in the half-life for sulfamethoxazole by seven days compared to 11.4 days for aerobic degradation of this antibiotic. In turn, no significant effects of incubation conditions on degradation rate were shown for trimethoprim, for which the calculated half-life was about 26 days under both aerobic and anaerobic conditions (Lin and Gan, 2011). The degradation of antibiotics may also be influenced by the pH and moisture content of soils. Weerasinghe and Towner (1997) showed that the half-life of for virginiamycin was negatively correlated with the pH of soils and ranged from 87 to 173 days for different agricultural soils. A study by Wang et al. (2006) revealed that the half-life of sulfadimethoxine in soil decreased from 10.4 days to 6.9, and again to 4.9 days when the soil moisture content was increased from 15, to 20, and 25%, respectively. A similar effect of temperature was shown in the case of the degradation of norfloxacin (Yang et al., 2012).

Degradation of antibiotics strongly depends on their concentration in soil. Increasing dosages of ciprofloxacin (from 1 to 5 and 50 mg/kg soil) led to a reduction of degradation from 75, to 62, and 40% within 40 days (Cui et al., 2014). A similar tendency was also shown by Demoling et al. (2009), who applied sulfamethoxazole at 500, 20, and 1 mg/kg soil and observed a reduction of its removal from 153, to 1.5, and 0.04 mg/kg after 5 weeks. The same trend was reported in degradation of azithromycin, ofloxacin, tetracycline (Walters et al., 2010), sulfadimethoxine (Wang et al., 2006), chlortetracycline (Fang et al., 2016), and SDZ (Zhang et al., 2017a). These results suggest that high concentrations of antibiotics may prolong their persistence in soils, due to the inhibition of the activity of soil microorganisms (Yang et al., 2009; Pan and Chu, 2016). However, application of unrealistically high concentrations tends to overestimate half-lives, which may not reflect realistic situations (Pan and Chu, 2016). The history of antibiotics application also plays a role in the further disappearance of antibiotics in soils. Repeated application of the macrolide antibiotics clarithromycin and erythromycin into soil resulted in a decrease of the DT50 values from 36.48 and 69.93 days to 15.85 and 4.36 days (soil with a history of clarithromycin and erythromycin application at 0.1 mg/kg soil) and to 9.51 and 0.94 days (soil with a history of clarithromycin and erythromycin application at 10 mg/kg soil), respectively (Topp et al., 2016). This phenomenon of pre-adaptation of soil microorganisms was also found for the degradation of chlortetracycline, sulfamethazine and tylosin (Topp et al., 2013).

Most published data have also indicated that the addition of different organic compounds, such as manure, biosolids, slurry, sludge, and compost, into soils may contribute to changes in the rate of antibiotics degradation (Table 3). Sassman and Lee (2007) showed that the addition of manure (20 mg/kg soil) increased the half-life of lasalocid from 3.6 to 4.3 days. In contrast, Yang et al. (2012) showed that norfloxacin (10 mg/kg soil) was degraded faster (half-life 24–39 days) in soil with manure (3–9%) compared to the control soil (half-life 48 days). In turn, Li et al. (2010a) found no significant effects from the addition of manure on degradation of chlortetracycline in soil.

## Impact of Antibiotics on Soil Microorganisms

Soil microorganisms perform many vital processes and participate in the maintenance of soil health and quality. They play a crucial role in organic matter turnover, nutrients release, and stabilization of the soil structure and ensure soil fertility. Moreover, many microorganisms act as biological control agents by inhibiting the growth of pathogens (Varma and Buscot, 2005). The homeostasis of soil may be disturbed by biotic and abiotic factors, including bacteriophages, predation, competition, pesticide, heavy metals, toxic hydrocarbons, and antibiotics (Cycoń et al., 2011; Cycoń and Piotrowska-Seget, 2015, 2016; Sułowicz and Piotrowska-Seget, 2016; Xu et al., 2016; Chen et al., 2017; Wepking et al., 2017; Orlewska et al., 2018a). The high antimicrobial activity of antibiotics in soil should differentially inhibit the growth of soil microorganisms and thus influence the soil microbial community composition, which may result in alterations of the ecological functionality of the soil (Kotzerke et al., 2008; Keen and Patrick, 2013; Molaei et al., 2017) (Figure 3).

There is abundant data on the impact of antibiotics on microorganisms and on soil processes mediated by bacteria and fungi. Both the effects of antibiotics on individual microbial populations as well as on the composition of entire microbial communities have been documented. A wide range of methods based on measurements of parameters that reflect the activity and abundance of total microbial communities, such as soil organic matter turnover, respiration, and microbial biomass have been used to characterize such effects. Other methods have focused on selected microbial processes such as nitrification, denitrification, sulfate, and iron reduction, methanogenesis and the activity of enzymes responsible for C, N, and P turnover (Brandt et al., 2009; Chen et al., 2013; Cui et al., 2014; Liu et al., 2015; Ma et al., 2016). A large number of studies have instead focused on assessing changes in microbial diversity, using metagenomics or 16S rRNA gene amplicon sequencing, as well as analysis of phospholipid fatty acids (PLFAs) isolated from soil (Zielezny et al., 2006; Hammesfahr et al., 2008; Reichel et al., 2015; Xu et al., 2016). In the following sections, the methods and parameters utilized to assess effects of antibiotics on the function and structure of soil microbial communities are presented and discussed (Figure 3).

## Impact of Antibiotics on Soil Microbe Function

### Soil Processes

Many studies have found that even low concentrations (below the MIC) of antibiotics influence various soil processes mediated by microorganisms (Table 4). A significant decrease in soil respiration (SR) was reported for soils containing sulfamethoxazole, sulfamethazine, sulfadiazine, and trimethoprim (Kotzerke et al., 2008; Liu et al., 2009), however, this effect was transient and depended on the disappearance rate of these antibiotics (DT50 2–5 days). The authors concluded that the effects of antibiotics on SR diminished due to decreased bioavailability of the antibiotic. In a study by Wepking et al. (2017), the response of microbial respiration to cephapirin, tetracycline, or erythromycin was dependent on both the type of antibiotic and the exposure of soil to dairy cattle manure. In other studies, no obvious effects of tetracycline, chlortetracycline, oxytetracycline, sulfadiazine, sulfapyridine, or tylosin on SR were observed (Thiele-Bruhn and Beck, 2005; Liu et al., 2009; Toth et al., 2011).

**Table 4:** Effects of the selected antibiotics on the microbial processes in soils with different characteristics.

**Antibiotic**	**Dosage (mg/kg soil)**	**Type of soil/origin**	**Main properties of soil**	**Experimental conditions**	**Effect (comparison with control, non-treated soil)**	**References**
	100	Loam (China)	Sand 42%, silt 38%, clay 20%, pH 6.31, OM 1.29%, WHC 35%	25°C, 50% WHC, 20 days	Basal respiration was stimulated	Ma et al., 2014
Ciprofloxacin	1, 5, and 50	Ustic Cambisol (China)	Sand 12%, silt 54%, clay 34%, pH 7.9, OC 36.76 g/kg, CEC 13.82 cmol/kg	25°C, 60% WHC, 40 days	Basal respiration was higher in treated soils at intermediate concentrations than in control at 9 and 22 days; nitrification was stimulated at 1 mg/kg and inhibited at 50 mg/kg after 9 days, nitrate and ammonium contents were not altered after antibiotic addition	Cui et al., 2014
	0.2, 2, and 20	Agricultural soil (Germany)	Sand 11%, silt 68%, clay 21%, pH 6.6, WHC 37.5%, OC 2.1%	20°C, 60% WHC, 93 days, sludge addition at 1.8 g/kg	Soil respiration was inhibited by ~70% at all three concentrations after 2 days and as only about 35% at the end of the experiment	Girardi et al., 2011
Chlortetracycline	1–50	Orthic Luvisol (Germany)	Sand 3%, silt 79%, clay 18%, pH 7, OC 1.04%, WHC 48.8%	20°C, 48 days	Basal respiration was not affected	Zielezny et al., 2006
	1–300	Silt loam (China)	OC 18.2 g/kg, pH 5,7	25°C, 60% WHC, 22 days	In general, no inhibitory effect on respiration was found	Liu et al., 2009
	0.0003–0.03	Silt loam (USA)	pH 6.5, OM 2.2%, WHC 0.313 g/g, CEC 10.6 cmol+/kg	80% WHC, manure addition, 50 days	No effect on nitrification, Fe(III) reduction or soil respiration	Toth et al., 2011
Difloxacin	1–100	Loamy sand (Germany)	Sand 73.3%, silt 23.1%, clay 3.6%, pH 5.5, OC 1.7%, WHC 27%	10°C, 60% WHC, manure addition at 40 ml/kg, 32 days	Increase rate of respiration up to 8 days; no detectable effect on ammonium and nitrate rates; higher nitrification in all treatments on day 8 and their reduction at 10 and 100 mg/kg on day 32; a significant lower potential denitrification on days 4 and 8 in all treatments	Kotzerke et al., 2011
Monensin	0.01–0.100	Silt loam (USA)	pH 6.5, OM 2.2%, WHC 0.313 g/g, CEC 10.6 cmol+/kg	80% WHC, manure addition, 50 days	Soil respiration was not affected; inhibition of the Fe(III) reduction was transient; low concentrations inhibited nitrogen transformation	Toth et al., 2011
Norfloxacin	5, 10, and 30	Acidic Soil (China)	Sand 29%, silt 39%, clay 32%, pH 4.3, OM 2.4%, CEC 9.5 cmol+/kg	25°C, 50% WHC, 42 days	No obvious inhibition on soil respiration, only slight effect on nitrogen transformation	Yang et al., 2012
Oxytetracycline	1–100	Agricultural soil (Iran)	Sand 52.35%, silt 29.23%, clay 18.42%, pH 4.3, OC 0.95%, WHC 20%	25°C, 50% WHC, 21 days	Impact on Fe(III) reduction at 1 and 10 mg/kg, and Fe(III) reduction was completely inhibited at concentrations above 10 mg/kg; negatively affected respiration throughout the experiment	Molaei et al., 2017
	1–30	Alfisol (China)	Sand 7.7%, silt 77.5%, clay 14.8%, pH 6.24, OM 2.4%, CEC 12.3 cmol/kg, OTC 37.3 μg/kg	25°C, 60% WHC, 120 days	Nitrification decreased over the experiment with transient increase on day 28	Ma et al., 2016
	10–1,000	Luvisol (Germany)	Sand 68.4%, silt 20.4%, clay 9.9%, pH 7.1, OC 1.6%, CEC 13.1 cmol/kg	20–25°C, 50% WHC, 14 days	No detectable effect on basal respiration; EC50 for Fe(III) reduction was 96 and 5.3 μg/g for Cambisol and Luvisol, respectively	Thiele-Bruhn and Beck, 2005
		Cmabisol (Germany)	Sand 80.9%, silt 15.9%, clay 3.1%, pH 6.6, OC 0.8%, CEC 5.3 cmol/kg			
Sulfadimethoxine	0.025–0.200	Silt loam (USA)	pH 6.5, OM 2.2%, WHC 0.313 g/g, CEC 10.6 cmol+/kg	80% WHC, manure addition, 50 days	Soil respiration was not affected; inhibition of soil nitrogen transformation; nearly completely blocked Fe(III) reduction throughout the 50-day experiment at higher concentrations (0.1 and 0.2 mg/kg)	Toth et al., 2011
Sulfadiazine	10 and 100	Silt loam (Germany)	Sand 6.4%, silt 78.2%, clay 15.4%, pH 7.2, OC 2.1%, WHC 46%	20°C, 32/61 days, manure addition at 40 g/kg soil	Only at 100 mg/kg higher ammonium and lower nitrate concentrations were detected; a reduction in CO_2_ production at the beginning of the experiment only in a higher treatment; increase and reduction of nitrification at 10 and 100 mg/kg, respectively after 32 days	Kotzerke et al., 2008; Schauss et al., 2009
		Loamy sand (Germany)	Sand 73.3%, silt 23.1%, clay 3.6%, pH 5.5, OC 1.7%, WHC 27%		Increased amounts of ammonium and reduced amounts of nitrate were determined in both treatments after 61 days; a reduction in CO_2_ production at the beginning of the experiment in both treatments; increase and reduction of nitrification at 10 and 100 mg/kg, respectively after 32 days	
	1–50	Orthic Luvisol (Germany)	Sand 3%, silt 79%, clay 18%, pH 7, OC 1.04%, WHC 48.8%	20°C, 48 days	Basal respiration was not affected	Zielezny et al., 2006
	10 and 100	Luvisol (China)	Sand 58.4%, silt 21.7%, clay 19.9%, pH 6.24, OM 3.56%, CEC 5.38 cmol/kg	25°C, 25% WHC, manure addition at 40 mg/kg, 28 days	Inhibition of basal respiration by both dosages on days 1 and 7; increase in basal respiration at 10 mg/kg after 28 days	Xu et al., 2016
		Endogleyic Cambisol (Germany)	Sand 73.3%, silt 23.1%, clay 3.6%, pH 4.8, OC 1%	10°C, 50% WHC, manure addition at 20, 40, and 80 g/kg, 32 days	No significant impact on the rate of SIR	Hammesfahr et al., 2011
				10°C, 60% WHC, manure addition at 40 g/kg, 57 days	Reduction of nitrification and N mineralization; increase of ammonification	Hammesfahr et al., 2011
Sulfamonomethoxine	100	Loam (China)	Sand 42%, silt 38%, clay 20%, pH 6.31, OM 1.29%, WHC 35%	25°C, 50% WHC, 20 days	Basal respiration was stimulated	Ma et al., 2014
Sulfamethoxazole	1–300	Silt loam (China)	OC 18.2 g/kg, pH 5,7	25°C, 60% WHC, 22 days	Decrease of respiration within the first 4 days; increase with increasing antibiotic concentrations after initial inhibition	Liu et al., 2009
	1–100	Agricultural soil (Iran)	Sand 52.35%, silt 29.23%, clay 18.42%, pH 4.3, OC 0.95%, WHC 20%	25°C, 50% WHC, 21 days	Affected Fe(III) reduction at 1 and 10 mg/kg, and Fe(III) reduction was completely inhibited at concentrations above 10 mg/kg; affected respiration at different treatments over experimental period	Molaei et al., 2017
Sulfamethazine	20 and 100	Silt loam (Korea)	pH 6.0, OM 2.36%	25°C, 70% WHC, 56 days, poultry manure addition (1%)	Increase of the CO_2_-C efflux during incubation time except for 56 day; increase of the nitrification at 28 and 56 days	Awad et al., 2016
Sulfapyridine	10–1,000	Luvisol (Germany)	Sand 68.4%, silt 20.4%, clay 9.9%, pH 7.1, OC 1.6%, CEC 13.1 cmol/kg	20–25°C, 50% WHC, 14 days	Basal respiration was not affected; EC50 for Fe(III) reduction was 12,400 and 310 μg/g for Cambisol and Luvisol, respectively	Thiele-Bruhn and Beck, 2005
		Cmabisol (Germany)	Sand 80.9%, silt 15.9%, clay 3.1%, pH 6.6, OC 0.8%, CEC 5.3 cmol/kg			
Tetracycline	100	Loam (China)	Sand 42%, silt 38%, clay 20%, pH 6.31, OM 1.29%, WHC 35%	25°C, 50% WHC, 20 days	Basal respiration was stimulated No inhibitory effect on respiration	Ma et al., 2014
Trimethoprim	1–300	Silt loam (China)	OC 18.2 g/kg, pH 5,7	25°C, 60% WHC, 22 days		Liu et al., 2009
					Decrease of respiration within the first 4 days; increase with increasing antibiotic concentrations after initial inhibition No inhibitory effect on respiration	
Tylosin	2,000	Sand (Denmark)	Sand 90.7%, silt 2.8%, clay 4.1%, pH 6.8, WHC 15%, OC 1.2%	25°C, 60 days	No significant effect on soil respiration	Müller et al., 2002

*CEC, cation exchange capacity; OC, organic carbon; OM, organic matter; SIR, substrate-induced respiration; WHC, water holding capacity*.

Nitrification and/or denitrification rates were also influenced by antibiotic exposure, and the effects were strongly dependent on the type of antibiotic and the length of exposure. For example, sulfadimethoxine inhibited soil nitrification; however, this effect was only observed on some sampling days and only for a high sulfadimethoxine treatment (Toth et al., 2011). A decrease in the nitrification rate caused by a high oxytetracycline concentration (30 mg/kg) and sulfadiazine (100 mg/kg) in a single application was also observed by Ma et al. (2016) and Kotzerke et al. (2008), whereas ciprofloxacin and norfloxacin were reported to stimulate the rate of nitrification in a soil microcosm, but only at the lowest concentration of antibiotic (1 mg/kg soil) (Yang et al., 2012; Cui et al., 2014). A study by DeVries et al. (2015) revealed that low doses of sulfamethoxazole, sulfadiazine, narasin, or gentamicin (500 μg/kg soil) inhibited denitrification, but dosages < 1 μg/kg soil actually stimulated the process transiently. Toth et al. (2011) did not observe any effects of monensin and chlortetracycline, on soil nitrification at concentrations of 0.01–0.1 and 0.0003–0.3 mg/kg soil, respectively.

Antibiotics may also change the turnover rate of iron in soil. Sulfadiazine and monensin blocked Fe(III) reduction in soil over periods ranging from a few days to the end of a 50-day experiment (Toth et al., 2011). Strong inhibition of Fe(III) reduction was also observed in soil contaminated with sulfamethoxazole and oxytetracycline at concentrations >10 mg/kg soil (Molaei et al., 2017). Thiele-Bruhn and Beck (2005) calculated the EC50 value of sulfapyridine for the microbial reduction of Fe(III) in two different soils at 12.4 and 0.310 mg/kg soil. It should be noted that the lack of standardized tests hinders comparisons that would lead to general conclusions about the effects of antibiotics on biogeochemical cycles and the turnover of iron.

### Enzyme Activities

Specific enzyme activity is considered to be a useful indicator of the response of microorganisms to stress caused in the soil by antibiotics (Unger et al., 2013; Liu et al., 2014, 2015; Ma et al., 2016) (Table 5). Enzyme activity indicates the potential of microbial communities to carry out biochemical processes that are essential to maintain soil quality. Any application of a toxicant that might affect the growth of soil microorganisms can induce alterations in the general activity of enzymes, such as dehydrogenases (DHAs), phosphatases (PHOSs), and urease (URE) (Gil-Sotres et al., 2005; Hammesfahr et al., 2011; Cycoń et al., 2016a). Inhibition of DHAs and URE was observed in soil amended with tetracycline at a concentration of 1 μg/kg soil, however this dosage did not affect acid phosphatase (PHOS-H) activity. Sulfamethazine applied at 53.6 μg/g soil had a significant short-term negative impact on the activities of DHAs and URE (Pinna et al., 2012). Inhibition of DHAs and arylsulphatase activities with increasing concentration of oxytetracycline at 1 to 200 mg/kg over 7 weeks was also reported by Chen et al. (2013). Benzylpenicillin, tylosin and sulfadiazine inhibited soil DHAs and PHOSs from 35 to 70% compared to the non-antibiotic amended control (Reichel et al., 2014; Akimenko et al., 2015). The results obtained by Unger et al. (2013) showed that the application of oxytetracycline or lincomycin (50 and 200 mg/kg soil) resulted in a temporary decrease in DHA activity in soil. A temporary increase, followed by a decrease, in DHA activity was found in soils containing chlortetracycline (1, 10, and 100 mg/kg soil) by Liu et al. (2015). In another study, DHA activity was unaffected by sulfapyridine or oxytetracycline, even at a dosage of 1 mg/kg soil (Thiele-Bruhn and Beck, 2005).

**Table 5:** Effects of the selected antibiotics on the enzyme activities in soils with different characteristics.

**Antibiotic**	**Dosage (mg/kg soil)**	**Type of soil/origin**	**Main properties of soil**	**Experimental conditions**	**Effect (comparison with control, non-treated soil)**	**References**
	1–300	Silt loam (China)	OC 18.2 g/kg, pH 5,7	25°C, 60% WHC, 22 days	Inhibition of acid PHOS during the 22-day experiment	Liu et al., 2009
Chlortetracycline	10 and 100	No data (China)	Sand 42.95%, silt 43.43%, clay 13.65%, pH 7.6, OC 20.7 g/kg	25°C, 50% WHC, 45 days, DOM addition at 40 mg C/kg	Inhibition of DHA until day 12; decrease of acid PHOS on days 6 and 12 at 10 and 100 mg/kg, respectively; inhibition of URE by 100 mg/kg on day 45	Liu et al., 2014
	1–100	No data (China)		25°C, 50% WHC, 45 days	Stimulation of all enzyme activities on the first day and then inhibition of DHA and URE up to 45 days; slight effect on PHOS	Liu et al., 2015
Lincomycin	5–200	Silt loam (USA)	Clay 23.4–26.2%, pH 6.7–7.3, OC 19.3–26.0 g/kg	25°C, 35% WHC, 63 days	Low DHA at 50 and 200 mg/kg and thereafter increase in all treatments; FDA at 50 and 200 mg/kg significantly higher on day 7	Unger et al., 2013
Oxyteracycline	1–200	Loam (China)	Sand 52.2%, silt 38.6%, clay 9.2%, pH 8.3, OM 1.2%	20–27°C (day) and 15–20°C (night), 50–70% WHC, manure addition at 30 mg/g, 7 weeks	DHA, ARYL, PHOS, and URE decreased with increasing concentrations of OTC	Chen et al., 2013
	5–200	Silt loam (USA)	Clay 23.4–26.2%, pH 6.7–7.3, OC 19.3–26.0 g/kg	25°C, 35% WHC, 63 days	DHA declined at 50 and 200 mg/kg up to day 35 and recovered on day 63; FDA at 200 mg/kg significantly higher on day 7 and recovered on day 14	Unger et al., 2013
	10–70	Wheat rhizosphere soil (China)	No data	30 days	Among alkaline PHOS, acid PHOS, DHA, and URE, only alkaline PHOS was higher at 10 mg/kg but further decreased at the dosage over 30 mg/kg	Yang et al., 2009
	1–30	Alfisol (China)	Sand 7.7%, silt 77.5%, clay 14.8%, pH 6.24, OM 2.4%, CEC 12.3 cmol/kg, OTC 37.3 μg/kg	25°C, 60% WHC, 120 days	Stimulation of DHA on day 14 and decrease by 120 days; no marked effect on PHOS and URE over the 120-day experiment	Ma et al., 2016
	10–1,000	Luvisol (Germany)	Sand 68.4%, silt 20.4%, clay 9.9%, pH 7.1, OC 1.6%, CEC 13.1 cmol/kg	20–25°C, 50% WHC, 14 days	No detectable effect on DHA	Thiele-Bruhn and Beck, 2005
		Cmabisol (Germany)	Sand 80.9%, silt 15.9%, clay 3.1%, pH 6.6, OC 0.8%, CEC 5.3 cmol/kg			
Penicillin G	100 and 600	Loamy soil (Russia)	pH 7.7, humus 4.1%, N_tot_ 0.25%, P 28.8, K_tot_ 2.06%	20–25°C, 60% WHC, 120 days	Inhibition of CAT, DHA, PHOS, and INV (20–70% of the control)	Akimenko et al., 2015
Sulfadimethoxine+sulfamethoxazole+sulfamethazine	0.09–900	Sandy loam (USA)	Sand 85.5%, silt 8.5%, clay 6.6%, pH 6.31, OC 0.86%, CEC 8.1 cmol+/kg	20°C, 21 days, glucose and/or manure addition	DHA and URE activities decreased with higher concentrations	Gutiérrez et al., 2010
Sulfadiazine	10 and 100	Luvisol (China)	Sand 58.4%, silt 21.7%, clay 19.9%, pH 6.24, OM 3.56%, CEC 5.38 cmol/kg	25°C, 25% WHC, manure addition at 40 mg/kg, 28 days	Inhibition of FDA by both dosages; DHA was significantly inhibited at 10 and 100 mg/kg within 14 days, a significant increase of DHA at 10 mg/kg after 28 days	Xu et al., 2016
		Endogleyic Cambisol (Germany)	Sand 73.3%, silt 23.1%, clay 3.6%, pH 4.8, OC 1%	10°C, 50% WHC, manure addition at 20, 40, and 80 g/kg, 32 days	No significant impact on β-GLU; declined URE	Hammesfahr et al., 2011
Sulfamethoxazole	1–300	Silt loam (China)	OC 18.2 g/kg, pH 5,7	25°C, 60% WHC, 22 days	Decline in acid PHOS during the 22-day experiment	Liu et al., 2009
	5	Silt loam (New Zeland)	Sand 9%, silt 54%, clay 37%, pH 6.7, OC 5%	25°C, 60% WHC, 36 days	DHA was not affected by antibiotic; an increase in the DHA	Srinivasan and Sarmah, 2014
		Clay loam (New Zeland)	Sand 13.7%, silt 51%, clay 30.4%, pH 5.8, OC 4%			
		Silt loam (New Zeland)	Sand 34%, silt 48%, clay 17%, pH 5.7, OC 8.2%			
Sulfamethazine	1–300	Silt loam (China)	OC 18.2 g/kg, pH 5,7	25°C, 60% WHC, 22 days	Inhibition of acid PHOS during 22 days	Liu et al., 2009
	53.6	Sandy loam (Italy)	Sand 72.7%, silt 10.6%, clay 16.6%, pH 7.8, OC 2.8%	7 days	A significant decrease of DHA (41%) and URE (38%) after 1 day and this effect disappeared after 7 days	Pinna et al., 2012
			Sand 81.7%, silt 5.9%, clay 12.2%, pH 5.3, OC 1.7%		A significant decrease of DHA (17%) and URE (27%) after 1 day and this effect disappeared after 7 days	
Sulfapyridine	10–1,000	Luvisol (Germany)	Sand 68.4%, silt 20.4%, clay 9.9%, pH 7.1, OC 1.6%, CEC 13.1 cmol/kg	20–25°C, 50% WHC, 14 days	No detectable effect on DHA	Thiele-Bruhn and Beck, 2005
		Cmabisol (Germany)	Sand 80.9%, silt 15.9%, clay 3.1%, pH 6.6, OC 0.8%, CEC 5.3 cmol/kg			
Tetracycline	1–300	Silt loam (China)	OC 18.2 g/kg, pH 5,7	25°C, 60% WHC, 22 days	Inhibition of acid PHOS during 22 days	Liu et al., 2009
	100 and 500	Clay (Italy)	Sand 39.4%, silt 19.2%, clay 41.4%, pH 5.8, OM 6.9%	20°C, 60 days	Decrease in FDA after 2 days; this effect disappeared after 7 days	Chessa et al., 2016b
		Sand (Italy)	Sand 72.7%, silt 10.6%, clay 16.6%, pH 7.6, OM 4.9%			
Trimethoprim	1–300	Silt loam (China)	OC 18.2 g/kg, pH 5,7	25°C, 60% WHC, 22 days	Inhibition of acid PHOS during 22 days	Liu et al., 2009
Tylosin	1–300	Silt loam (China)	OC 18.2 g/kg, pH 5,7	25°C, 60% WHC, 22 days	Decline in acid PHOS during the 22-day experiment	Liu et al., 2009
	100 and 600	Loamy soil (Russia)	pH 7.7, humus 4.1%, N_tot_ 0.25%, P 28.8, K_tot_ 2.06%	20–25°C, 60% WHC, 120 days	Suppressing effect on CAT, DHA, PHOS, and INV (20–70% of the control)	Akimenko et al., 2015
Vancomycin	1 and 10	Sandy loam (Poland)	Sand 67%, silt 24%, clay 9%, pH 6.9, WHC 43%, OC 1.6%, CEC 10 cmol/kg	22°C, 50% WHC, 90 days	A negative impact on days 1, 15, and 30 as was showed by a decrease in the values of DHA, PHOS and URE (6–32%)	Cycoń et al., 2018

*ARYL, arylsulfatase; CAT, catalase; CEC, cation exchange capacity; DHA, dehydrogenase; FDA, fluorescein diacetate; β-GLU, β-glucosidase; INV, invertase; OC, organic carbon; OM, organic matter; PHOS, phosphatase; URE, urease; WHC, water holding capacity*.

Various effects on the activity of PHOSs from different antibiotics applied to soil have been shown. For example, six antibiotics, i.e., chlortetracycline, tetracycline, tylosin, sulfamethoxazole, sulfamethazine, and trimethoprim at dosages of 1–300 mg/kg soil inhibited the activity of PHOS-H (Liu et al., 2009, 2015). In contrast, results from Yang et al. (2009) indicated that only alkaline phosphatase (PHOS-OH) was sensitive to the application of oxytetracycline, with a 41.3% decline in enzyme activity at a concentration of 10 mg/kg soil and a further decrease of 64.3–80.8% when the dose of the antibiotic exceeded 30 mg/kg. Ma et al. (2016) found that OTC at dosages up to 30 mg/kg soil had no effect on the activity of neutral PHOS over a 120-day experimental period.

The inhibition of enzyme activity in antibiotic-treated soils may be related to inhibition of growth or death of sensitive microorganisms (Boyd and Mortland, 1990; Gianfreda et al., 1994; Alef, 1995; Marx et al., 2005). In turn, the increased activity of enzymes under antibiotics pressure may result from the ability of many bacteria to subsist on antibiotics as a carbon source (Dantas et al., 2008). We can speculate that enzymes produced by such bacteria compensate for the negative effects of antibiotics on enzyme activity by increasing the activity of the antibiotic-resistant microbial community. Similarly, an increase in the activity of some enzymes in soils treated with different antibiotics might be related to the capability of some microorganisms to make use of the antibiotics in their metabolisms, thus resulting in an increase in the abundance of some soil microorganisms and enzyme production. In addition, the presence of some antibiotics in soil may cause an overgrowth of fungi, which are generally less sensitive to antibiotics than bacteria. Fungi are a major producer of enzymes in soils and so may be responsible for observed increases in enzyme activity (Tabatabai and Bremner, 1969; Westergaard et al., 2001; Hammesfahr et al., 2011; Ding et al., 2014).

### Functional Capacity Evaluation

Functional microbial diversity reflects the ability of an entire microbial community to utilize a suite of substrates (Zak et al., 1994). The response of microorganisms to the presence of antibiotics in soils, expressed as the community level physiological profile (CLPP), can be evaluated using the Biolog (EcoPlates™) or MicroResp™ methods, which are based on the utilization of 31 or 15 carbon sources, respectively, by organisms present in soil extracts (Toth et al., 2011; Ma et al., 2014: Xu et al., 2016) (Table 6). Although the exact numbers and taxonomic identities of the bacterial species responsible for the Biolog reactions remain uncertain, patterns of functional diversity indicate how biodiversity affects specific ecosystem functions (Laureto et al., 2015). For determination of CLPP, the biodiversity (Shannon H') and average well-color development (AWCD) indices have been used as indicators of changes in the catabolic potential of soil microorganisms exposed to antibiotics. For example, chlortetracycline (1 and 10 mg/kg soil) caused a decrease in AWCD values, showing low catabolic potential in the microbial community, however, this effect was seen only at the beginning of the experiment. After 35 days, irrespective of the frequency of chlortetracycline application, the AWCD values gradually recovered to the control level (Fang et al., 2014, 2016). A slight inhibitory effect on microbial activity (expressed as the H′ index) in soil was observed over a range from 1 to 300 mg oxytetracycline /kg soil. Even lower concentrations of this antibiotic (0.5 to 90 mg/kg of soil) significantly decreased the functional activity (expressed as AWCD) of the soil microbial communities (Kong et al., 2006). Sulfonamide antibiotics, such as sulfamethoxazole and sulfamethazine, can also alter the activity of microbial populations, however, they were observed to only have short-term detrimental effects (Demoling et al., 2009; Pinna et al., 2012; Pino-Otín et al., 2017). In turn, the application of chlortetracycline or sulfadimethoxine to soil did not significantly change AWCD, whereas monensin slightly increased the value of the H' index (Toth et al., 2011). In many studies, changes in the preferential degradation of groups of substrates in Eco-plates, e.g., amino acids, carbohydrates, carboxylic acids, aromatic acids, and miscellaneous, by microorganisms were observed over the course of an experimental period. Xu et al. (2016) found that a high sulfadiazine concentration decreased utilization rates of amino acids, carbohydrates, carboxylic acids, and aromatic acids by bacteria present in a soil suspension. A significant decrease in the utilization of carbohydrates and miscellaneous substrates was also observed in soil that had been treated with sulfamethoxazole (Liu et al., 2012a). However, this effect was only observed 7 days after the antibiotic application; by day 21 substrate utilization had increased compared to the first sampling day. The negative impact of antibiotics on CLPP may be mitigated by the enrichment of soil with organic matter from animal wastes, antibiotic-free manure or plant wastes (Demoling et al., 2009; Pinna et al., 2012; Liu et al., 2014). Contrarily, Wang et al. (2016) found that doxycycline application at 10 mg/kg soil increased utilization of the substrates.

**Table 6:** Effects of the selected antibiotics on the community-level physiological profile (CLPP) in soils with different characteristics.

**Antibiotic**	**Dosage (mg/kg soil)**	**Type of soil/origin**	**Main properties of soil**	**Experimental conditions**	**Effect (comparison with control, non-treated soil)**	**References**
Ciprofloxacin	100	Loam (China)	Sand 42%, silt 38%, clay 20%, pH 6.31, OM 1.29%, WHC 35%	25°C, 50% WHC, 20 days	Functional diversity decreased	Ma et al., 2014
Cefuroxime	1 and 10	Sandy loam (Poland)	Sand 67%, silt 24%, clay 9%, pH 6.9, WHC 43%, OC 1.6%, CEC 10 cmol/kg	22°C, 50% WHC, 90 days	A negative impact of CEF up to 30 days	Orlewska et al., 2018a
	1–300	Clay loam (China)	Sand 30.4%, silt 34.1%, clay 35.5%, pH 5.7, OC 18.2 g/kg, CEC 9.87 cmol/kg	25°C, 60% WHC, 21 days	No effect on AWCD and Shannon indices after 7 days; a significant increase of AWCD at 300 mg CTC/kg after 21 days	Liu et al., 2012a
Chlortetracycline	1 and 100	Inceptisol (China)	Sand 21.5%, silt 71.1%, clay 7.4%, pH 6.8, OM 3.1%, WHC 27%, CEC 10.6 cmol/kg	25°C, 60% WHC, 35 days	AWCD and functional diversity indices decreased significantly up to 35 days	Fang et al., 2016
	10 and 100	No data (China)	Sand 42.95%, silt 43.43%, clay 13.65%, pH 7.6, OC 20.7 g/kg	25°C, 50% WHC, 45 days, DOM addition at 40 mg C/kg	Decrease in AWCD up to 45%; a significantly detrimental effect on the diversity and richness of the microbial community with maximum decrease of 4 and 21%, respectively	Liu et al., 2014
	1–100	No data (China)	Sand 42.95%, silt 43.43%, clay 13.65%, pH 7.6, OC 20.7 g/kg	25°C, 50% WHC, 45 days	AWCD, richness, and Shannon indices were higher with maximum values of 56.5, 42.9, and 13.9%, respectively; no significant difference on days 6 and 12, with the exception of a larger value in the highest treatment to the end of the experiment	Liu et al., 2015
	0.0003–0.03	Silt loam (USA)	pH 6.5, OM 2.2%, WHC 0.313 g/g, CEC 10.6 cmol+/kg	80% WHC, manure addition, 50 days	CLPP parameters, including AWCD, Shannon diversity index, and evenness was not affected	Toth et al., 2011
Doxycycline	10	Silty clay loam (China)	Sand 9%, silt 63.7%, clay 27.3%, pH 7, OM 1%	25°C, 50–65% WHC, manure addition at 10 g/kg, 56 days	Positively affected on the microbial diversity	Wang et al., 2016
Monensin	0.01–0.100	Silt loam (USA)	pH 6.5, OM 2.2%, WHC 0.313 g/g, CEC 10.6 cmol+/kg	80% WHC, manure addition, 50 days	4–5% increase in Shannon diversity index in 0.025, 0.05, and 0.100 mg/kg treatments	Toth et al., 2011
Oxytetracycline	1–217	Silt loam (Anthroposols) (China)	No data	25°C	Functional diversity, evenness, AWCD and substrate utilization decreased significantly with increasing concentrations	Kong et al., 2006
	1–200	Loam (China)	Sand 52.2%, silt 38.6%, clay 9.2%, pH 8.3, OM 1.2%	Room temperature, 50–60% WHC, manure addition at 30 mg/g, 6 days	AWCD values increased, and the utilization of sugar and its derivatives enhanced	Liu et al., 2012b
	5–200	Silt loam (USA)	Clay 23.4–26.2%, pH 6.7–7.3, OC 19.3–26.0 g/kg	25°C, 35% WHC, 63 days	Increase of AWCD, richness, and diversity at 50 mg/kg on day 7	Unger et al., 2013
	1–30	Alfisol (China)	Sand 7.7%, silt 77.5%, clay 14.8%, pH 6.24, OM 2.4%, CEC 12.3 cmol/kg, OTC 37.3 μg/kg	25°C, 60% WHC, 120 days	Negative effect on soil microbial community metabolism, but not on functional diversity indices	Ma et al., 2016
Sulfadimethoxine	0.025–200	Silt loam (USA)	pH 6.5, OM 2.2%, WHC 0.313 g/g, CEC 10.6 cmol+/kg	80% WHC, manure addition, 50 days	CLPP parameters, including AWCD, Shannon diversity index, and evenness were not affected	Toth et al., 2011
Sulfadiazine	10 and 100	Luvisol (China)	Sand 58.4%, silt 21.7%, clay 19.9%, pH 6.24, OM 3.56%, CEC 5.38 cmol/kg	25°C, 25% WHC, manure addition at 40 mg/kg, 28 days	High dosage decreased the utilization rates of four categories of substrates (carbohydrate, carboxylic acid, amino acid, and aromatic acid) and the values of Shannon index during the experiment, while no significant inhibition was found at lower dosage on day 28	Xu et al., 2016
Sulfamonomethoxine	100	Loam (China)	Sand 42%, silt 38%, clay 20%, pH 6.31, OM 1.29%, WHC 35%	25°C, 50% WHC, 20 days	Negative effect on functional diversity; more than half of the CLPP substrates could not be utilized effectively in soils	Ma et al., 2014
Sulfamethoxazole	20 and 500	Loamy sand (Netherlands)	Sand 78.9%, silt 10.4%, clay 7%, pH 4.9, OC 3.7%	25°C, 35% WHC, 5 weeks	Negative effect on CLPP at the highest concentration	Demoling et al., 2009
	10	Silty clay loam soil (China)	Sand 9%, silt 63.7%, clay 27.3%, pH 7, OM 1%	25°C, 50–65% WHC, manure addition at 10 g/kg, 56 days	An inhibitory effect on the microbial diversity	Wang et al., 2016
	100 and 1,000	No data (Spain)	Sand 37.3%, silt 24.7%, clay 38.0%, pH 7.9, OM 3.9%	22–25°C, 70% WHC, 14 days	Decrease in AWCD and physiological diversity; changes in pattern of substrate utilization (decrease in all substrates at both concentrations, except polymers, and amino acids)	Pino-Otín et al., 2017
	1–100	Clay loam (China)	Sand 30.4%, silt 34.1%, clay 35.5%, pH 5.7, OC 18.2 g/kg, CEC 9.87 cmol/kg	25°C, 60% WHC, 21 days	Decrease in functional diversity after 7 days; 100 mg/kg decreased AWCD and Shannon indices; enhanced soil microbial community function on day 21	Liu et al., 2012a
Sulfamethazine	53.6	Sandy loam (Italy)	Sand 72.7%, silt 10.6%, clay 16.6%, pH 7.8, OC 2.8%	7 days	AWCD decreased after 1 day and increased after 7 days; richness increased during the experiment	Pinna et al., 2012
			Sand 81.7%, silt 5.9%, clay 12.2%, pH 5.3, OC 1.7%		AWCD decreased after 1 day and increased after 7 days; richness decreased during the experiment	
Tetracycline	100 and 500	Clay (Italy)	Sand 39.4%, silt 19.2%, clay 41.4%, pH 5.8, OM 6.9%	20°C, 60 days	Microbial communities in both soils were affected in the short term 500 mg/kg soil	Chessa et al., 2016b
		Sand (Italy)	Sand 72.7%, silt 10.6%, clay 16.6%, pH 7.6, OM 4.9%			
	100	Loam (China)	Sand 42%, silt 38%, clay 20%, pH 6.31, OM 1.29%, WHC 35%	25°C, 50% WHC, 20 days	Functional diversity decreased	Ma et al., 2014
	100 and 1,000	No data (Spain)	Sand 37.3%, silt 24.7%, clay 38.0%, pH 7.9, OM 3.9%	22–25oC, 70% WHC, 14 days	Decrease in physiological diversity; changes in pattern of substrate utilization (decrease in carboxylic and ketonic acids and increase in amines/amides at 100 mg/kg	Pino-Otín et al., 2017
Trimethoprim					Decrease in physiological diversity; changes in pattern of substrate utilization (decrease in carboxylic and ketonic acids and increase in polymers at 100 mg/kg)	
Tylosin	2,000	Sand (Denmark)	Sand 90.7%, silt 2.8%, clay 4.1%, pH 6.8, WHC 15%, OC 1.2%	25°C, 15% WHC, 60 days	No differences in the number of substrates utilized	Westergaard et al., 2001
					No differences in the functional diversity patterns	Müller et al., 2002
Vancomycin	1 and 10	Sandy loam (Poland)	Sand 67%, silt 24%, clay 9%, pH 6.9, WHC 43%, OC 1.6%, CEC 10 cmol/kg	22°C, 50% WHC, 90 days	A negative impact on days 1, 15, and 30 as was showed by a decrease in the values of the CLPP indices (10–69%)	Cycoń et al., 2018

*AWCD, average well-color development; CEC, cation exchange capacity; CLPP, community-level physiological profile; OC, organic carbon; OM, organic matter; WHC, water holding capacity*.

CLPP patterns do not always show changes in the functional diversity of microorganisms in response to antibiotic. In such situations, the lack of observable effects in CLPP might be because indices expressing total microbial activity do not detect possible changes caused by low antibiotic concentrations. Moreover, repeated application of antibiotics leads to the adaptation of bacteria to these compounds and proliferation of antibiotic-resistant microorganisms. Another limitation is that the Biolog technique measures only the activity of catabolically active cells and omits non-culturable populations as well as microorganisms in a dormant state. Moreover, fast-growing microorganisms are mainly responsible for effects observed by this test (Floch et al., 2011).

## Impact of Antibiotics on Microbial Community Structure

### PLFA Fingerprinting

The PLFA method is a rapid tool for assessing the biomass and composition of microbial communities in soil, because various phospholipid-derived fatty acids (PLFAs) are indicative of species or microbial groups in soil. In addition, the ratios of biomass of bacteria/fungi and Gram-positive/Gram-negative bacteria are often used to evaluate the response of microorganisms to organic pollutants (Frostegård et al., 1993, 2011).

In general, application of antibiotics into soil show strong and dose-dependent effects on the microbial structure expressed as the total PLFA biomass (Table 7). For example, Chen et al. (2013) found a significant increase in bacterial and fungal PLFA biomass 7 weeks after oxytetracycline contamination of 1 and 15 mg/kg soil, but a strong decrease in the case of 200 mg antibiotic/kg treatment. A decrease in microbial PLFA biomass content was also found in soils that had been treated with penicillin G (10 and 100 mg/kg soil) and tetracycline (100 mg/kg soil) (Zhang et al., 2016a). Simultaneously, the ratios of Gram-negative/Gram-positive bacteria increased, indicating that the bacteria that were resistant to antibiotics in tested soils were more likely to be Gram-negative. A study by Unger et al. (2013) found only short-term effects (35 days) from lincomycin or oxytetracycline applications (5–200 mg/kg soil), suggesting that the resilience of soil microbial communities toward effects of these antibiotics.

**Table 7:** Effects of the selected antibiotics on the community structure of microorganisms based on the PLFA analysis in soils with different characteristics.

**Antibiotic**	**Dosage (mg/kg soil)**	**Type of soil/origin**	**Main properties of soil**	**Experimental conditions**	**Effect (comparison with control, non-treated soil)**	**References**
Ciprofoxacin	1, 5, and 50	Ustic Cambisol (China)	Sand 12%, silt 54%, clay 34%, pH 7.9, OC 36.76 g/kg, CEC 13.82 cmol/kg	25°C, 60% WHC, 40 days	Decrease in the ratio of bacteria to fungi and increased in the ratio of Gram+ to Gram-bacteria, PCA of the PLFA data clearly distinguished among different concentrations	Cui et al., 2014
Difloxacin	1–100	Loamy sand (Germany)	Sand 73.3%, silt 23.1%, clay 3.6%, pH 5.5, OC 1.7%, WHC 27%	10°C, 60% WHC, manure addition at 40 ml/kg, 32 days	Impact on the total PLFAs only on days 1 and 8; a significant decrease in the ratio of bacteria/fungi on day 8 at 100 mg/kg; a significant decrease in the ratio of Gram+/Gram– on day 1 after application regardless of the concentration	Kotzerke et al., 2011
	0.452	Luvisol (Germany)	Sand 6%, silt 78%, clay 16%, pH 6.3, OC 1.2%, CEC 11.4 cmol/kg	21°C, 63 days, slurry addition at 40 ml/kg	The total PLFAs increased on days 7 and 14; temporal shifts to the Gram– bacteria indicated by decrease in the ratio of Gram+/Gram– on days 14 and 63; shifts to fungi shown by lower ration of bacteria/fungi on days 42	Reichel et al., 2013
Lincomycin	5–200	Silt loam (USA)	Clay 23.4–26.2%, pH 6.7–7.3, OC 19.3–26.0 g/kg	25°C, 35% WHC, 63 days	All PLFA markers declined between 0 and 3 days; decline in total biomass, Gram+, Gram–, anaerobic bacteria, fungi, and mycorrhizae between 3 and 35 days; increase in the total biomass, bacteria/fungi ratio, Gram–, and anaerobic bacteria and protozoa between 35 and 63 days	Unger et al., 2013
Oxytetracycline	1–200	Loam (China)	Sand 52.2%, silt 38.6%, clay 9.2%, pH 8.3, OM 1.2%	20–27°C (day) and 15–20°C (night), 50–70% WHC, manure addition at 30 mg/g, 7 weeks	The total PLFAs increased at 1 at 15 mg/kg and decreased at 200 mg/kg; fungal PLFAs were significantly lower at 200 mg/kg; the ratios of Gram–/Gram+ at 1 and 15 mg/kg were significantly higher while at 200 mg/kg was significantly lower	Chen et al., 2013
	5–200	Silt loam (USA)	Clay 23.4–26.2%, pH 6.7–7.3, OC 19.3–26.0 g/kg	25°C, 35% WHC, 63 days	All PLFA markers declined between 0 and 3 days; additional declines in biomass, Gram+, Gram–, anaerobic bacteria, fungi, and mycorrhizae between 3 and 35 days; decline in Gram+, fungi, and mycorrhizae and increase in biomass, ratio of bacteria/fungi, Gram–, and anaerobic bacteria and protozoa between 35 and 63 days	Unger et al., 2013
Sulfadimethoxine+ Sulfamethoxazole+ Sulfamethazine	0.09–900	Sandy loam (USA)	Sand 85.5%, silt 8.5%, clay 6.6%, pH 6.31, OC 0.86%, CEC 8.1 cmol+/kg	20°C, 21 days, glucose and/or manure addition	A relative community shift toward Gram- bacteria and increase in fungal biomass	Gutiérrez et al., 2010
Sulfadiazine		Luvisol (China)	Sand 58.4%, silt 21.7%, clay 19.9%, pH 6.24, OM 3.56%, CEC 5.38 cmol/kg	25°C, 25% WHC, manure addition at 40 mg/kg, 28 days	The total PLFA, bacterial, and actinomycetes biomass was reduced; no significant effect on the ratio of Gram+/Gram– bacteria; decrease in the ratio of bacteria/fungi	Xu et al., 2016
	10 and 100	Loamy sand (Germany)	Sand 73.3%, silt 23.1%, clay 3.6%, pH 4.8, OC 1%	10°C, 50% WHC, manure addition at 20, 40, and 80 g/kg, 32 days	Decrease in the total PLFAs on days 1 and 8 and on day 32 for antibiotic in combination with high and low manure amendment; decrease in the ratio of bacteria/fungi for SDZ with low manure amendment on day 32; no effect on the ration of Gram+/Gram– bacteria	Hammesfahr et al., 2011
				10°C, 50% WHC, manure addition at 40 mg/g, 61 days	Decrease of the effect of manure on the total of PLFAs at all day	Hammesfahr et al., 2008
		Silt loam (Germany)	Sand 6.4%, silt 78.2%, clay 15.4%, pH 7.2, OC 2.1%		SDZ decreased the effect of manure on the total of PLFAs at all days	
	0.256	Silt loam (Germany)	Sand 6%, silt 78%, clay 16%, pH 6.3, OC 1.2%, CEC 11.4 cmol/kg	21°C, 63 days, slurry addition at 40 ml/kg	The total PLFAs increased on day 63; temporal shifts to the Gram– bacteria shown by the decrease in the ratio of Gram+/Gram- on days 14 and 63; shifts to fungi characterized by lower ratio of bacteria/fungi on day 14	Reichel et al., 2013
	1 and 10			21°C, 63 days, manure addition	Decrease in the total PLFAs by 14% in the rhizosphere and 3% in bulk soil in the field experiment	Reichel et al., 2014
	20 and 500	Loamy sand (Netherlands)	Sand 78.9%, silt 10.4%, clay 7%, pH 4.9, OC 3.7%	25°C, 35% WHC, 5 weeks	No differences in the microbial community structure	Demoling et al., 2009
		Silt loam (New Zeland)	Sand 9%, silt 54%, clay 37%, pH 6.7, OC 5%			
Sulfamethoxazole	5	Clay loam (New Zeland)	Sand 13.7%, silt 51%, clay 30.4%, pH 5.8, OC 4%	25°C, 60% WHC, 36 days	A higher proportion of bacterial biomass over fungal biomass (fungal to bacterial ratio < 1) for each sampling event	Srinivasan and Sarmah, 2014
		Silt loam (New Zeland)	Sand 34%, silt 48%, clay 17%, pH 5.7, OC 8.2%			
Tetracycline	100 and 500	Clay (Italy)	Sand 39.4%, silt 19.2%, clay 41.4%, pH 5.8, OM 6.9%	20°C, 60 days	Short-term negative effect at a higher concentration; a significant increase in the ratio of fungi: bacteria in both soil, and Gram+/Gram– in clay soil	Chessa et al., 2016b
		Sand (Italy)	Sand 72.7%, silt 10.6%, clay 16.6%, pH 7.6, OM 4.9%			
Vancomycin	1 and 10	Sandy loam (Poland)	Sand 67%, silt 24%, clay 9%, pH 6.9, WHC 43%, OC 1.6%, CEC 10 cmol/kg	22°C, 50% WHC, 90 days	A temporal shift and dominance of Gram-; a decrease in the ratio of Gram+/Gram–; increase in fungal biomass as reflected by decreased ration of bacteria/fungi	Cycoń et al., 2016a

*OC, organic carbon; OM, organic matter; PLFA, phospholipid fatty acid; WHC, water holding capacity*.

An increase in total PLFA biomass was also observed in soils treated with sulfadiazine and difloxacin in slurry from pigs medicated with these antibiotics (Reichel et al., 2013). Influences from the difloxacin-slurry on PLFA biomass were observed at the beginning of the experiment (7 and 14 days), but only on the last sampling day (63 day) in the sulfadiazine-slurry. The authors hypothesized that the delayed effect of sulfadiazine was a result of continuous remobilization of antibiotic residues bound to soil particles by microorganisms. Hammesfahr et al. (2008) and Kotzerke et al. (2011), in contrast, found no changes in soil microbial communities upon addition of the above-mentioned antibiotics into soil spiked with slurry.

Several studies showed changes in the soil fungal PLFA signatures upon amendment with antibiotics. For example, the content of fungal marker fatty acids (18:1ω9 and 18:2ω6,9) significantly increased in alfalfa-amended soil and both alfalfa- and manure-amended soils with 20 and 500 mg sulfamethoxazole /kg of soil (Demoling et al., 2009). Gutiérrez et al. (2010) mixed three sulfonamides, sulfadimethoxine, sulfamethoxazole, and sulfamethazine, and observed a general increase in the proportion of fungal PLFAs among the total soil biomass PLFAs. A shift in the soil microbial community toward more fungi because of sulfapyridine application was also noted by Thiele-Bruhn and Beck (2005). The increase in the abundance of fungi may ultimately result in a decline in the productivity and quality of soils and agricultural products (Ding and He, 2010).

The PLFA method, previously used extensively for determination of microbial community composition, has been largely replaced by techniques based on the analysis of nucleic acids extracted from soil. However, PLFA-based analyses remain an adequately sensitive, efficient way to rapidly screen whether a microbial community has been affected by antibiotics.

### Phylogenetic Analyses

Many authors have used PCR-dependent DNA fingerprinting techniques such as a terminal restriction fragment length polymorphism (T-RFLP) or denaturing gradient gel electrophoresis (DGGE) to evaluate the impact of antibiotics on diversity of soil microorganisms (Müller et al., 2002; Reichel et al., 2014; Chessa et al., 2016a; Orlewska et al., 2018a,b). Most of these studies target 16S rRNA genes and show altered diversity upon antibiotics applications. These changes may be explained by the disappearance of sensitive bacteria and outgrowth of resistant bacteria present in the antibiotic-contaminated soils (Table 8). For example, the application of tylosin (2 mg/kg soil) strongly modified the DGGE pattern, reducing the number of bands observed on days 15 and 22 of the experiment compared to non-exposed soil. However, on day 33 the difference was smaller, yet detectable (Westergaard et al., 2001). In a study by Müller et al. (2002), the DGGE pattern of 16S rDNA in tylosin-treated soil differed slightly to the diversity to the control soil, although colony morphology typing and potential of microbial communities to utilize selected substrates did not reveal any differences. Cycoń et al. (2016a) found that vancomycin dosed to soil at 10 mg/kg resulted in quantitative changes in microbial community patterns, suggesting selection exerted by the antibiotic. The changes in DGGE patterns suggest a decrease in populations of a species or group of species; revealing that some bacterial species were more sensitive to vancomycin than others were. This was corroborated by a decline in the values of H' and S (richness) indices. Another study showed significant effects of chlortetracycline on the microbial community that lasted for no longer than 1 week after the application of the antibiotic as the communities recovered over time until the end of the experiment (42 day) (Nelson et al., 2011). Contrarily, using PCR-DGGE Zielezny et al. (2006) showed that the application of sulfadiazine and chlortetracycline at concentrations of 1, 10, and 50 mg/kg soil had no significant effect on the structure of the soil microbial community. In another study, changes in DGGE profiles showed that sulfadiazine (10 and 100 μg/g soil) applied to soil in manure altered bacterial diversity (Hammesfahr et al., 2008). Although the DGGE profiles proved the impact of manure and sulfadiazine on the total soil bacterial communities, these effects were less distinct for pseudomonads and β-Proteobacteria. This observation may be explained by the resistance of many strains to sulfonamides, which may be only indirectly affected by sulfadiazine. Genetic changes within the β-Proteobacteria and the pseudomonads, expressed by the appearance or loss of a band in the DGGE profiles from manure, and sulfadiazine-amended soils were also reported by Reichel et al. (2014). However, statistical analysis showed that the β-Proteobacteria significantly responded to the time and moisture regimes, whereas the pseudomonads responded to the factors of time and treatment. The effects of sulfadiazine-containing manure on microbial diversity resulted in a low stability of soil bacterial communities and dominance of taxa that are known to contain human pathogens, such as *Gemmatimonas, Leifsonia, Devosia, Clostridium, Shinella*, and *Peptostreptococcus* (Ding et al., 2014). No apparent effects on microbial diversity in response to streptomycin and difloxacin were observed in similar studies (Shade et al., 2013; Jechalke et al., 2014a).

**Table 8:** Effects of the selected antibiotics on the community structure of microorganisms based on the genetic analyses in soils with different characteristics.

**Antibiotic**	**Dosage (mg/kg soil)**	**Type of soil/origin**	**Main properties of soil**	**Experimental conditions**	**Effect (comparison with control, non-treated soil)**	**References**
Amoxycillin	10 and 100	Silt loam (Germany)	pH 7.2, WHC 46%, OC 2.1%	10°C, 40% WHC, manure addition at 40 ml/kg, 9 days	Transient modification in the composition of bacterial community revealed by DGGE patterns	Binh et al., 2007
		Loamy sand (Germany)	pH 5.5, WHC 27%, OC 1.7%			
Cefuroxime	1 and 10	Sandy loam (Poland)	Sand 67%, silt 24%, clay 9%, pH 6.9, WHC 43%, OC 1.6%, CEC 10 cmol/kg	22°C, 50% WHC, 90 days	Alteration in the DGGE profiles and decline in the DGGE indices (richness and diversity) at 10 mg/kg soil, in particular on days 30, 60, and 90	Orlewska et al., 2018c
Ciprofloxacin	100	Loam (China)	Sand 42%, silt 38%, clay 20%, pH 6.31, OM 1.29%, WHC 35%	25°C, 50% WHC, 20 days	Lower abundance of six nitrogen-cycling genes including *chiA, amoA, nifH, nirK, nirS*, and *narG* in the treated soil	Ma et al., 2014
	0.2, 2, and 20	Agricultural soil (Germany)	Sand 11%, silt 68%, clay 21%, pH 6.6, WHC 37.5%, OC 2.1%	20°C, 60% WHC, 93 days, sludge addition at 1.8 g/kg	Shift in both microbial abundance and microbial diversity based on the T-RFLP analysis	Girardi et al., 2011
Chlortetracycline	1–50	Orthic Luvisol (Germany)	Sand 3%, silt 79%, clay 18%, pH 7, OC 1.04%, WHC 48.8%	20°C, 48 days	No detectable impact on the community structure showed by DGGE pattern	Zielezny et al., 2006
Difloxacin	0.452	Luvisol (Germany)	Sand 6%, silt 78%, clay 16%, pH 6.3, OC 1.2%, CEC 11.4 cmol/kg	21°C, 63 days, slurry addition at 40 ml/kg	Changes in DGGE profiles; additionally, clusters of the antibiotic-slurry treatment were less strongly shifted by time	Reichel et al., 2013
	5	Silt loam (Germany)	Sand 6.4%, silt 78.2%, clay 15.4%, pH 7.2, OC 2.1%, WHC 46%	Field experiment, 140 days	Significant effect of antibiotic- manure on the bacterial community composition was revealed by DGGE; quinolone resistance genes *qnrB* and *qnrS1*/*qnrS2* were detected by PCR and subsequent hybridization, while *qnrA* was not detected; PCA of DGGE profiles suggested that effect of manure lasted till day 28; samples on days 71 and 140 were closed to untreated soil	Jechalke et al., 2014b
Erythromycin	1 and 10	Sandy loam (Poland)	Sand 67%, silt 24%, clay 9%, pH 6.9, WHC 43%, OC 1.6%, CEC 10 cmol/kg	22°C, 50% WHC, 90 days	Alteration in the DGGE profiles and decline in the DGGE indices (richness and diversity) at 10 mg/kg soil, in particular on days 15, 30, and 60	Orlewska et al., 2018b
Lincomycin	0.05 and 5	Cambisol (Czech Republic)	Sand 17%, silt 57%, clay 26%, pH 7.65, OM 8.17%	16/6°C, 40 days	Dosage of 5 mg/kg shifted bacterial diversity (16S rRNA gene—T-RFLP) in the soil with high pH, while in the soil with low pH higher percentage of actinomycetes and higher diversity of the *lmrB* homolog genes did not change; sequencing of 157 new clones of *lmrB* homologs revealed selection of *lmrB* homologs in both soils	Cermák et al., 2008
		Podzol (Czech Republic)	Sand 96%, silt 2%, clay 2%, pH 4.01, OM 9.08%			
Sulfachloropyridiazine	1, 10, and 100	Silt Loam (US)	Sand 19.9%, silt 56.6%, clay 23.6%, pH 7.5, OC 1.8	25°C, 40 days	No significant differences in banding patterns (Dice similarity coefficients above 0.9)	Accinelli et al., 2007
		Sand (US)	Sand 93.5%, silt 2.7%, clay 3.8%, pH 7.2, OC 0.94%			
Sulfadiazine	20	Silt loam (Germany)	Sand 6.4%, silt 78.2%, clay 15.4%, pH 7.2, OC 2.1%, WHC 46%	20°C, 30 days, 50% WHC, manure addition	Contamination of the manure with antibiotic significantly reduced *nifH*, ammonia-oxidizing bacteria (AOB) *amoA, nirK, nirS*, and *nosZ* gene abundance patterns 20 days after application, however ammonia-oxidizing archaea (AOA), *amoA* gene abundance were not influenced at any sampling day	Ollivier et al., 2010
	10 and 100	Silt loam (Germany)	Sand 6.4%, silt 78.2%, clay 15.4%, pH 7.2, OC 2.1%, WHC 46%	20°C, 61 days, manure addition at 40 g/kg soil	High prevalence of *sul1* in manure and manured soils, *sul2* was mainly found in the loamy sand treated with manure and high dose and *sul3* was not detected; addition of manure increased *nirK*-harboring denitrifiers in both soils, whereas in the treatments the abundance of the *nirS* denitrifiers increased after the bioavailable antibiotic had declined; however, the community composition of *nirS* nitrite reducers investigated by DGGE did not change despite the observed alterations in abundance.	Heuer and Smalla, 2007; Schauss et al., 2009; Kleineidam et al., 2010; Heuer et al., 2011b
		Loamy sand (Germany)	Sand 73.3%, silt 23.1%, clay 3.6%, pH 5.5, OC 1.7%, WHC 27%			
	1–50	Orthic Luvisol (Germany)	Sand 3%, silt 79%, clay 18%, pH 7, OC 1.04%, WHC 48.8%	20°C, 48 days	Modification in the composition of a bacterial community shown by DGGE	Zielezny et al., 2006
	10 and 100	Loamy sand (Germany)	Sand 73.3%, silt 23.1%, clay 3.6%, pH 5.5, OC 1%	10°C, 50% WHC, manure addition at 40 mg/g, 61 days	Increase in the band intensity in DGGE profiles in manure treatment, while manure, and antibiotic had a more differentiated effect on bacterial populations; changes in DGGE profiles were seen even after two month of the experiment	Hammesfahr et al., 2008
		Silt loam (Germany)				
	10 and 100	Silt loam (China)	No data	15°C, 55% WHC, manure addition at 40 g/kg, 193 days	Repeated application at a higher concentration of 100 mg/kg soil caused visible changes in the composition of a bacterial community	Ding et al., 2014
	0.256	Silt loam (Germany)	Sand 6%, silt 78%, clay 16%, pH 6.3, OC 1.2%, CEC 11.4 cmol/kg	21°C, 63 days, slurry addition at 40 ml/kg	Changes in the total DGGE band profiles; the samples of control- and antibiotic-slurry treatment clustered separately mostly by time	Reichel et al., 2013
	1 and 10			21°C, 63 days, manure addition	DGGE pattern indicated larger structural shifts within genus of *Pseudomonas* in SDZ-contaminated earthworm burrows compared to bulk soils	Reichel et al., 2014
Sulfamonmethoxine	100	Loam (China)	Sand 42%, silt 38%, clay 20%, pH 6.31, OM 1.29%, WHC 35%	25°C, 50% WHC, 20 days	Lower abundances of six nitrogen-cycling genes including *chiA, amoA, nifH, nirK, nirS*, and *narG* were observed	Ma et al., 2014
Sulfamethazine	1, 10, and 100	Silt Loam (US)	Sand 19.9%, silt 56.6%, clay 23.6%, pH 7.5, OC 1.8	25°C, 40 days	No significant t differences in banding patterns (Diece similarity coefficients above 0.9)	Accinelli et al., 2007
		Sand (US)	Sand 93.5%, silt 2.7%, clay 3.8%, pH 7.2, OC 0.94%			
Tetracycline	100	Loam (China)	Sand 42%, silt 38%, clay 20%, pH 6.31, OM 1.29%, WHC 35%	25°C, 50% WHC, 20 days	Lower abundances of six nitrogen-cycling genes including *chiA, amoA, nifH, nirK, nirS*, and *narG* were revealed	Ma et al., 2014
Tylosin	2,000	Sand (Denmark)	Sand 90.7%, silt 2.8%, clay 5.1%, pH 6.8, WHC 15%, OC 1.2%	25°C, 15% WHC, 60 days	Decrease in the number of bands in DGGE profiles was detected on days 15 and 22; difference was smaller but still detectable on day 33 and at the end of experiment	Westergaard et al., 2001
					Small difference in the microbial diversity shown by DGGE	Müller et al., 2002
Vancomycin	1 and 10	Sandy loam (Poland)	Sand 67%, silt 24%, clay 9%, pH 6.9, WHC 43%, OC 1.6%, CEC 10 cmol/kg	22°C, 50% WHC, 90 days	Disappearance of some bands in DGGE profiles; decrease of the overall richness and diversity of the dominant bacteria at 10 mg/kg during the 90-day experiment	Cycoń et al., 2016a

*DGGE, denaturing gradient gel electrophoresis; OC, organic carbon; OM, organic matter; T-RFLP, terminal restriction fragment length polymorphisms; WHC, water holding capacity*.

## Diversity of Antimicrobial Resistance Genes

**Figure 5:**
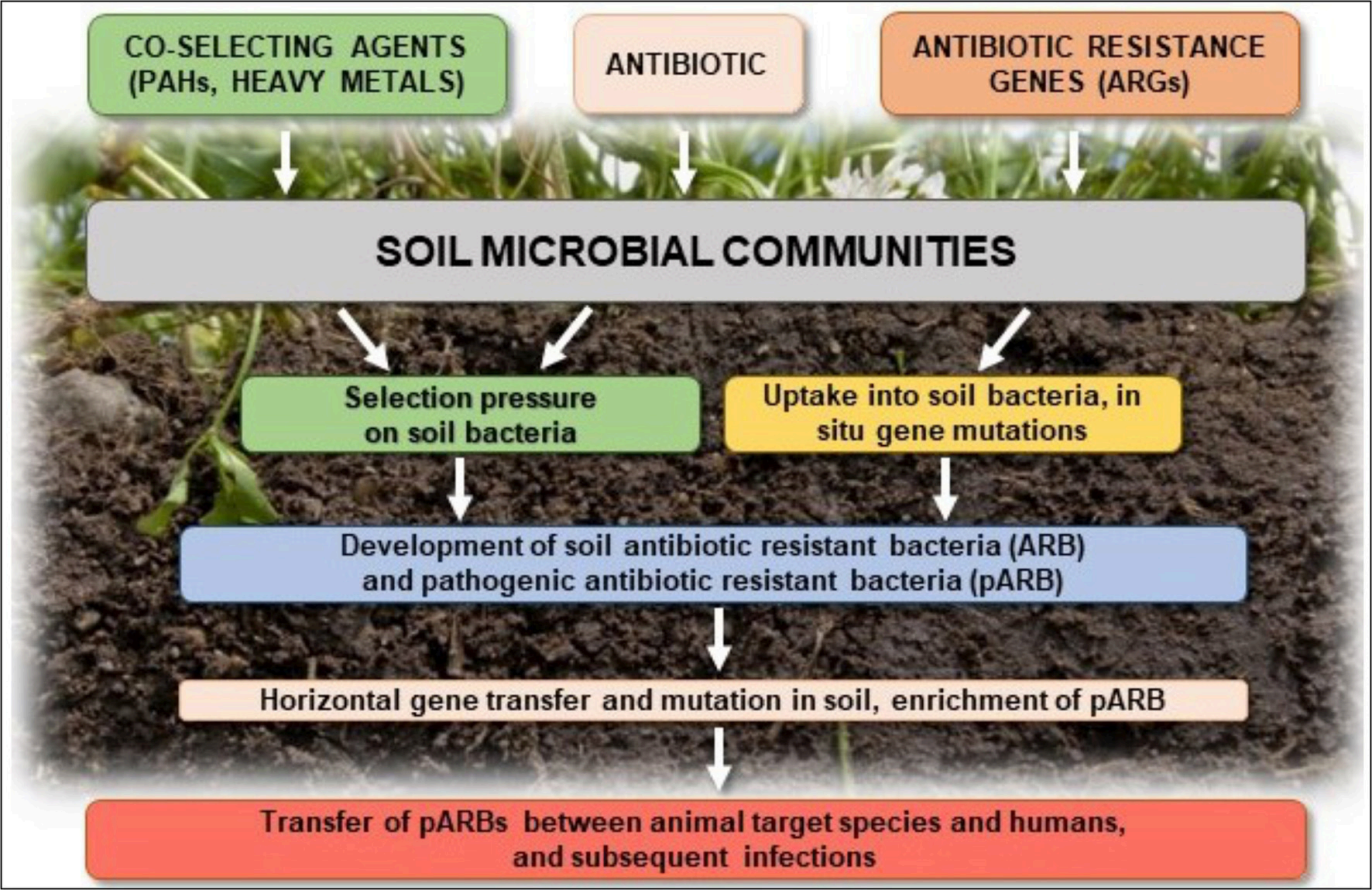
Transfer of antibiotic resistance in soil and risk of animal and human infection (based on Ashbolt et al., 2013).

Studies are increasingly focusing on genes for antimicrobial resistance itself, which can be followed by metagenomic sequencing approaches. Resistance determinants present in the soil, defined as the soil resistome, include genes that confer antibiotic resistance to pathogenic and non-pathogenic species present in the environment, as well as proto-resistance genes that serve as a source of resistance elements (D'Costa et al., 2006, 2007; Perry et al., 2014). The potential transfer of antibiotic resistance among microbial populations in soil and the risk of animal and human infection are major concerns (Figure 5).

Special attention is given to the detection and quantitation of ARGs, identifying resistance mechanisms, discovery of novel enzymes with unexpected activities, and the impact of pollutants and agricultural practices on the abundance of ARGs in soils. Resistance to antibiotics has been documented even in bacteria inhabiting pristine soils never exposed to these compounds, for example genes conferring resistance to common antibiotics (β-lactams, fluoroquinolones, and tetracyclines) were found in the Tibet (Chen et al., 2016). Interestingly, a tiny fraction of ARGs located on MGEs has been discovered, indicating a low, but real, potential for these ARGs to be transferred among bacteria (Chen et al., 2016). Additionally, some ARGs that have been discovered encode efflux pumps that are completely different from previously known efflux pumps. A large number (177) of ARGs encoding mostly single or multi-drug efflux pumps conferring resistance to aminoglycosides, chloramphenicol and β-lactams were reported in pristine Antarctic soils (Van Goethem et al., 2018). A low number and diversity of ARGs was detected in glacial soil and permafrost as compared to environments highly impacted by human activities in a study by Zhang et al. (2018). It has been proposed that ARGs in pristine environments most likely represent functional historical genes conferring resistance to natural antibiotics. Antibiotic resistance appeared to be transferred vertically over generations with limited to no horizontal transfer of ARGs between species (Van Goethem et al., 2018).

Diversity and abundance of ARGs is strongly influenced by agricultural practices (Wepking et al., 2017; Xiong et al., 2018). It has been documented that the spreading of manure from animals treated with different antibiotics on fields affects the abundance and diversity of ARGs (Su et al., 2014; Kyselková et al., 2015). However, it has also been documented that organic matter content, pH, and the history of soil management are important factors influencing the fate and abundance of ARGs in the soil (Cermák et al., 2008; Popowska et al., 2012).

The genes conferring resistance to minocycline, tetracycline, streptomycin, gentamycin, kanamycin, amikacin, chloramphenicol, and rifampicin in soils subjected to manure application accounted for ~70% of the total ARGs. More than half of the ARGs shared low similarity, < 60%, with the sequences of their closest proteins in GeneBank (Su et al., 2014). Among the ARGs studied (*ampC, tetO, tetW*, and *ermB*), the average abundance of *ampC* and *tetO* in the manure-treated soil was 421 and 3.3% greater in comparison with a non-treated control soil, respectively (Wepking et al., 2017). A broad-spectrum profile of ARGs has been found in 5 paddy soils of South China (Xiao et al., 2016). Based on Antibiotic Resistance Genes Database and next generation sequencing, 16 types of ARGs corresponding to 110 ARG subtypes were identified. Multi-drug resistance genes dominated in all soils (up to 47.5%), followed mainly by specific resistance genes for acriflavine, macrolide-lincosamide-streptogramin and bacitracin. Efflux pumps, antibiotic deactivation, and cellular protection were three major resistance mechanisms inferred from the resistome characterized Xiao et al. (2016).

An increase in the number and proportions of ARGs in the bacterial community was also shown by Nõlvak et al. (2016), who studied the effect of animal manure on the rate of the dissemination of ARGs (*sul1, tetA, blaCTX-M, blaOXA2*, and *qnrS*) and integron-integrase genes in soil. The abundance of genes for tetracycline resistance (*tetM, tetO*, and *tetW*) in soil that had had manure applied was significantly higher than in untreated soil (Xiong et al., 2018). It seems that an increasing abundance of ARGs resulted from inputs of ARGs already present in manure. This is because antibiotics may provide a significant selective advantage for some species or groups of antibiotic-resistant bacteria that then come to dominate the microbial community.

By contrast, many studies have shown that the dissemination of ARGs in soils is more correlated with the application of manure than with the presence of antibiotics. Though no effect on the abundance of *tetG, tetO*, and *tetW* was observed in one study, despite the presence of these genes in manure applied to the soil (Fahrenfeld et al., 2013), other studies have found an impact of manure from never antibiotic-treated animals on the number, diversity, and spread potential of ARGs (Udikovic-Kolic et al., 2014; Hu et al., 2016). This phenomenon can be explained by the stimulation of growth autochthonous bacteria and accelerated spread of ARGs by fertilizers free from antibiotics. Due to the ambiguous results of studies concerning the spread of ARGs in manure-treated soil, there is a need for future research to clearly assess the relative contribution of manure and antibiotics on microbial community structure and processes mediated by soil microorganisms.

The studies on the fate of ARGs in soil allowed not only to assess the diversity and abundance of genes of interest, but also to discover new ARGs, and described new resistance mechanisms and novel enzymes responsible for resistance of bacteria to antibiotics (Lau et al., 2017; Wang et al., 2017). Functional metagenomics for the investigation of antibiotic resistance led to the discovery of several tetracycline resistance genes from different classes in soil. Efflux pump genes, including 21 major facilitator superfamily efflux pump genes, were found to dominate the sequence libraries derived from studied soils. In addition, two new genes involved in the enzymatic inactivation of tetracycline were identified (Wang et al., 2017). Similarly, Torres-Cortés et al. (2011) described a new type of dihydrofolate reductase (protein Tm8-3; 26.8 kDa) conferring resistance to trimethoprim in soil microbiome. Using functional metagenomics they also identified three new antibiotic resistance genes conferring resistance to ampicillin, two to gentamicin, two to chloramphenicol, and four to trimethoprim in libraries generated from three different soil samples. Nine carbapenem-hydrolizing metallo-beta-lactamases (MBLs) including seven novel enzymes showing 33–59% similarity to previously described MBLs were discovered. Six originated from *Proteobacteria*, two from *Bacteroidetes*, and one from *Gemmatimonadetes*. MBLs were detected more frequently and exhibited higher diversity in grassland than in agricultural soil (Gudeta et al., 2016). In a study of resistome of bacterial communities in Canadian soils by functional metagenomics, 34 new ARGs with high homology to determinants conferring resistance aminoglycosides, sulfonamides and broad range of β-lactams were identified (Lau et al., 2017). High-resolution proteomics in combination with functional metagenomics resulted in the discovery of a new proline-rich peptide PPP^AZI^ promoting resistance to various macrolides, but not to other ribosome-targeting antibiotics (Lau et al., 2017). Similarly, Donato et al. (2010) identified two novel bifunctional proteins responsible for bacteria resistance to ceftazidime and kanamycin, respectively.

Other synthetic pollutants can also influence the acquisition and maintenance of ARGs that can confer resistance not only to antibiotics but also to a number of structurally unrelated contaminants present in soil. As an example for this, ARGs were ~15 times more abundant in polycyclic aromatic hydrocarbon (PAH) contaminated soils than in similar non-contaminated soils (Chen et al., 2017). It resulted from the selection by PAHs of Proteobacteria containing ARGs encoded multi-drug efflux pumps leading to simultaneously enriching of ARGs carried by them in the soils. Moreover, most of ARGs (70%) found in the PAHs-contaminated soils were not carried by plasmids, indicating a low possibility of horizontal gene transfer (HGT) between bacteria (Chen et al., 2017).

It has recently been reported that heavy metals are also as a factor contributing to maintenance of ARGs in the environment. A study of dairy farms showed significant correlations between the abundance of ARGs, metal resistance genes and the content of Cu and Zn in cattle feces (Zhou et al., 2016). The role of heavy metals can be explained by cross-resistance, tolerance of bacteria to more than one antimicrobial agent (Chapman, 2003; Baker-Austin et al., 2006), and co-resistance, the localization of ARGs and genes encoding resistance to heavy metals and other antibacterial agents on mobile genetic elements (Chapman, 2003), mechanisms. Multi-drug efflux pumps, which mediate rapid extrusion of antibiotics and heavy metals from the cell decrease susceptibility toward these compounds, are an example of cross-resistance (Martinez et al., 2009). Co-resistance can occur due to the close arrangement of genes on a chromosome or extrachromosomal element, which increases the likelihood that genes are subject to combined transmission via HGT. Moreover, heavy metals not only trigger co-selection processes, but also increase the level of tolerance to antibiotics due to co-regulation of resistance genes (Baker-Austin et al., 2006). Such mechanisms may affect the spread of ARGs from environmental populations to bacteria of clinical importance even in the absence of direct antibiotics selection (Herrick et al., 2014).

Antibiotics concentrations in the environment are much lower than that used in therapeutic doses. However, even at sub-inhibitory concentrations (below the MIC), antibiotics may select for resistant phenotypes (Gullberg et al., 2011, 2014). It has been reported that resistant bacteria can be selected at antibiotic concentrations even 100-fold below lethal concentrations. Using data on MICs from the EUCAST database for 111 antibiotics, Bengtsson-Palme and Larsson (2016) estimated that the predicted no-effect concentrations (PNECs) range from 8 ng/L to 64 μg/L. Mutants selected by antibiotics at minimum selective concentration appeared to be more fit than those selected at high concentration and still highly resistant (Gullberg et al., 2011; Sandegren, 2014). Antibiotics at such concentrations play multifaceted roles in the soil, including influencing inter-species competition, signal communication, host–parasite interactions, virulence modulation, and biofilm formation (Sengupta et al., 2013). Moreover, they induce mutations, genetic recombination, and HGT processes (Andersson and Hughes, 2012, 2014). Sub-lethal concentrations of drugs can also increase the mutation rate and potential for enrichment of stable genetic mutants by the induction of SOS responses, increasing translation misreading, and generation of oxygen radicals. It has been reported that sub-lethal concentrations of tetracycline stimulated up to 1,000-fold higher horizontal transfer rates of mobile genetic elements in *Listeria monocytogenes* (Bahlm et al., 2004) and integron recombination rates in *Vibrio cholerae* (Guerin et al., 2009). However, the rate of spread of ARGs depends on the antibiotic concentration in the soil. Huang et al. (2016) showed that the dissipation rate of plasmid-located genes [i.e., *qnrS, oqxA, aac(6*′*)-Ib-cr*] was significantly lower in soil treated with ciprofloxacin at concentrations of 0.04 and 0.4 mg/kg soil compared to soil treated with antibiotic at 4 mg/kg and controls. Forsberg et al. (2012) also found little evidence for HGT in soil.

Because of the risks posed by the dissemination of ARGs among bacteria and plants, studies investigating new practices designed to decrease the accumulation and transport of ARGs within soil are being conducted. For example, the use of biochar as a soil amendment significantly reduced the abundance of tetracyclines (*tetC, tetG, tetW*, and *tetX*) and sulfonamides (*sulI* and *sulII*) resistance genes in soil and lettuce tissues (Ye et al., 2016; Duan et al., 2017).

## Conclusion

Environmental antibiotic pollution is a problem that is expected to gain more attention in the near future since antibiotic consumption is still increasing around the world. A review of available literature shows that transformation and/or degradation are the most important processes that determine the fate of antibiotics in soils and that soil microorganisms play an important role in these processes. However, the rate of these transformation and degradation depends largely on the antibiotic structure and is affected by many abiotic and biotic factors, as is most evident in the large range of DT50 values of antibiotics in different soils. Studies have shown that within groups of similar antibiotics or even particular antibiotics, DT50 values differ significantly because of various soil properties, antibiotic dosage, and environmental conditions.

Current literature reviewed here indicates that the input of antibiotics into soil alters the structure and activity of microbial communities and the abundance of ARGs. However, results from studies in this field are often ambiguous, making environmental risk assessments related to the presence of antibiotics depend upon too many different factors to be reliable. Interactions between soil, antibiotics, and microorganisms are multifarious and many environmental factors may influence the value of tested parameters. The effect of antibiotics on the activity and diversity of microbial communities depends on the physicochemical parameters of the soil, the antimicrobial activity, and dosage of the antibiotic, as well as the time of exposure. It has become clear that microorganisms that are sensitive to different antibiotics are killed or inhibited in the presence of antibiotics, which may result in outgrowth of resistant bacteria resistant. In turn, this may alter the diversity of the soil microbial communities. Contrarily, there is some evidence that certain microorganisms can adapt to and possibly transform antibiotics. The less toxic transformation products would favor recovery of the original microbial communities from the initial disturbances caused by antibiotics exposure. Several studies found transient negative effects of antibiotics on the functional, structural and genetic diversity of soil microbial communities; a temporary loss of soil functionality with subsequent recovery.

Existing OECD and ISO ecotoxicological tests used to evaluate the toxicity of chemicals on the activity of a single species (e.g., Microtox, MARA, measurements of selected enzyme activities) and some of the processes mediated by soil microorganisms are not sufficient to gauge the effects of antibiotics on soil microbial communities. Therefore, ecotoxicological tests that combine various microbial community parameters should be developed. Moreover, future studies should be based on “omics” approaches such as genomics, transcriptomics, and proteomics, that allow for deeper examination of microbial communities than CLPP, PLFA, and PCR-DGGE methods. “Omics” methods are better tools for tracking the fate and determining dissipation rate of ARGs, as well as for recognizing new mechanisms of antibiotic resistance. Future frameworks for antibiotics should focus on the effect of soil properties on the maintenance and development of antibiotic resistance, the fate of antibiotics and ARGs in manure-fertilized soils, the rate of HGT between antibiotic-resistant and autochthonous bacteria, the link between antibiotic concentrations and genetic changes in soil resistomes, and the role of various contaminants and co-selecting agents on maintenance of ARGs in soil. Moreover, there is a need to complement biological assays with advanced analytical methods (such as LC-MS/MS, isotope dilution mass-spectrometry) and proper sample preparation, allowing assessment of antibiotic concentrations and, especially, of bioavailability in complex environmental matrices. Other problems that should be taken into consideration are variations on the procedures for estimating the limits of antibiotic detection and the lack of standard analytical methods for monitoring antibiotic in the environment. Future studies should also be focused on design and management practices that minimize the spread of ARGs from harvested crops in antibiotic-treated soils into the food chain. Finally, though it is rarely studied, the genetic potential of microorganisms involved in the degradation of antibiotics in soil warrant wider investigation.

## Author Contributions

MC, AM, and ZP-S conceived and designed content of the paper and wrote the paper. MC prepared the figures and tables.

### Conflict of Interest Statement

The authors declare that the research was conducted in the absence of any commercial or financial relationships that could be construed as a potential conflict of interest. The reviewer UNR and handling Editor declared their shared affiliation.
